# Asymmetric Fe–Te Pairs Enhance Peroxymonosulfate Activation via Surface‐Bound Hydroxyl Radicals Pathways

**DOI:** 10.1002/anie.3343591

**Published:** 2026-05-02

**Authors:** Xuheng Li, Chunli Wang, Yuntong Sun, Sheng Wang, Min Zheng, Tierui Zhang, Jong‐Min Lee

**Affiliations:** ^1^ School of Chemistry and Chemical Engineering Xi'an University of Architecture and Technology Xi'an Shaanxi China; ^2^ School of Chemical Engineering Faculty of Sciences Engineering and Technology The University of Adelaide Adelaide South Australia Australia; ^3^ School of Chemistry Chemical Engineering and Biotechnology Nanyang Technological University Singapore Singapore; ^4^ State Key Laboratory of Water Pollution Control and Green Resource Recycling Shanghai Institute of Pollution Control and Ecological Security School of Environmental Science and Engineering Tongji University Shanghai China; ^5^ Key Laboratory of Photochemical Conversion and Optoelectronic Materials Technical Institute of Physics and Chemistry Chinese Academy of Sciences Beijing China; ^6^ Department of Energy Science and Engineering Daegu Gyeongbuk Institute of Science and Technology (DGIST) Daegu Republic of Korea; ^7^ Energy Science and Engineering Research Center Daegu Gyeongbuk Institute of Science and Technology (DGIST) Daegu Republic of Korea

**Keywords:** asymmetric Fe–Te dual‐atom, Fenton‐like, *p–d* hybridization, peroxymonosulfate, surface‐bound hydroxyl radicals

## Abstract

Controlling peroxymonosulfate (PMS) activation at the atomic scale is crucial for steering reactive oxygen species (ROS) pathways, yet design principles that selectively bias PMS chemistry toward interfacial radical states remain elusive. Herein, we report an asymmetric Fe–Te dual‐atom pair (FeTe DAs/NC), in which a p‐block metalloid electronically modulates an Fe center through pronounced *p–d* hybridization. This atomic asymmetry reconstructs the local electronic structure, strengthens PMS binding, and directs PMS activation toward the generation and retention of surface‐bound hydroxyl radicals. Mechanistic studies reveal surface‐bound hydroxyl radicals (^•^OH) as the dominant ROS, while singlet oxygen (^1^O_2_) plays a secondary role. As a result, FeTe DAs/NC achieves complete degradation of carbamazepine within 60 min, markedly outperforming Fe or Te single‐atom analogs, together with excellent reactivity and cycling stability across different water matrices and pollutant systems. This work establishes atomic‐scale asymmetry and metal–metalloid p–d coupling as an effective strategy for steering PMS activation chemistry toward long‐lived interfacial radical states.

## Graphical abstract

An asymmetric Fe–Te dual‐atom pair directs peroxymonosulfate activation toward long‐lived surface‐bound hydroxyl radicals through strong *p–d* hybridization. The resulting electronic asymmetry enables efficient pharmaceutical degradation and reveals a metal–metalloid pairing strategy for steering reactive oxygen species pathways in advanced oxidation catalysis.

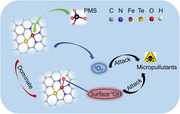

## 1 Introduction

Pharmaceutical and personal care products (PPCPs) have emerged as a class of persistent organic micropollutants in aquatic environments, raising serious concerns due to their chemical stability and potential risks to ecosystems and human health [1, 2]. Conventional water‐treatment processes are often ineffective in eliminating these trace contaminants, highlighting the urgent need for advanced oxidation technologies. Recently, peroxymonosulfate (PMS)‐based advanced oxidation processes (AOPs) have attracted increasing attention as a promising strategy for the degradation and mineralization of PPCPs [3]. The unique asymmetric structure of PMS (H─O─O─SO_3_^−^) endows it with dual redox properties, allowing it to act as both an electron donor and an electron acceptor. Upon activation, PMS can generate a variety of reactive oxygen species (ROS), including radical species (sulfate radicals (SO_4_^•−^) and hydroxyl radical (^•^OH), as well as non‐radical species (high valent metal‐oxo (M(IV) = O) and singlet oxygen (^1^O_2_) [4]. Among these, ^•^OH (*E*_0_ = 2.8 V vs. NHE) [5] and SO_4_^•−^ (*E*_0_ = 2.5–3.1 V vs. NHE) [6] exhibit higher oxidation potentials than M(IV) = O (E_0_> 1.95 V vs. NHE) [7] and ^1^O_2_ (*E*_0_ = 1.5 V vs. NHE) [8], making them highly effective for the rapid oxidation and mineralization of organic pollutants. However, their ultrashort half‐life spans (1 µs for ^•^OH and 30–40 µs for SO_4_^•−^) severely limit the probability of contact with target molecules, thereby reducing overall oxidation efficiency [4]. Enhancing ROS utilization efficiency, therefore, requires not only efficient PMS activation but also stabilization of the generated radicals and sustained interaction between ROS and pollutants. In this context, surface‐bound radicals have recently been recognized as key intermediates due to their extended lifetimes and spatially confined reactivity, which ensure efficient pollutant degradation while minimizing radical loss. Such behavior can be achieved by regulating the electron structure or spatial configuration of catalysts [9, 10], thereby imposing stringent requirements on catalyst design.

In recent years, atomically dispersed transition‐metal catalysts supported on carbon materials (especially Fe, Co, Cu, and Mn single atoms) have been widely investigated for PMS activation [11, 12, 13]. These catalysts feature high atomic utilization efficiency, well‐defined active sites, and tunable electronic structures. Among them, dual‐atom catalysts (DACs) have attracted increasing attention because they can overcome the linear adsorption limitations of isolated single sites [14, 15], thereby enabling the regulation of specific ROS generation pathways and improving selectivity and utilization efficiency [16]. For example, Fe–Co DACs can regulate the PMS adsorption configuration to achieve nearly 100% electron‐transfer efficiency for pollutant degradation [17], while Fe–Ni DACs coupled with N‐vacancy engineering facilitate electron transfer and accelerate reaction kinetics. Such enhancements are generally attributed to the electronic and synergistic effects in DACs, where the introduction of a second metal atom modulates the electronic distribution and simultaneously activates different elementary reaction steps, thus significantly improving catalytic activity [18, 19, 20, 21, 22].

Despite this progress, most reported DACs have primarily focused on promoting ^1^O_2_ formation, generating mixed radical systems, or enhancing electron‐transfer pathways, while offering limited regulation over radical stability. The stabilization of radicals depends not only on the electron‐donating and electron‐accepting properties of the active sites but also on the matching of their orbital energy levels [3, 13]. Introducing atoms with distinct orbital characteristics into dual‐atom configurations can lead to the formation of new hybridized orbitals, thereby reconstructing the electronic distribution and bonding characteristics of the active centers. Such orbital hybridization effects are expected to generate surface hydroxyl radicals with prolonged lifetimes and enhance the utilization efficiency of radicals without compromising catalytic activity. In particular, *p–d* orbital coupling between metals from different groups can effectively control the formation and cleavage of coordination bonds during dynamic catalytic processes [23, 24, 25]. However, it remains a challenge to identify suitable p‐block heterometal modulators capable of inducing effective *p–d* orbital hybridization for establishing more flexible electron‐transfer pathways and stable surface‐adsorbed radicals, thereby promoting the generation and conversion efficiency of reactive oxygen species (ROS).

Herein, we introduce the p‐block metalloid Te as a second atom adjacent to Fe single‐atom sites (SAs), forming an asymmetric Fe–Te dual‐atom pair (FeTe DAs/NC). These asymmetric pairs endow the active centers with enhanced redox tunability and superior PMS activation capability. The resulting FeTe DAs demonstrate exceptional performance in degrading micropollutants, highlighting their potential for practical water treatment applications. Combined quenching experiments, electron paramagnetic resonance (EPR) spectroscopy, in situ characterizations, and density functional theory (DFT) calculations reveal that surface‐bound ^•^OH is the primary reactive species, while ^1^O_2_ plays a secondary role. Notably, *p–d* orbital hybridization induces electron delocalization around the Fe center, modulating the desorption behavior of ^•^OH and favoring the formation of stabilized surface‐bound states. This work establishes a generalizable strategy for designing asymmetric metal‐metalloid pairs with controlled ^•^OH desorption properties, offering a new paradigm for high‐efficiency micropollutant degradation in PMS‐based advanced oxidation systems.

## 2 Results and Discussion

### 2.1 Construction of Asymmetric Fe–Te Pairs

**FIGURE 1:**
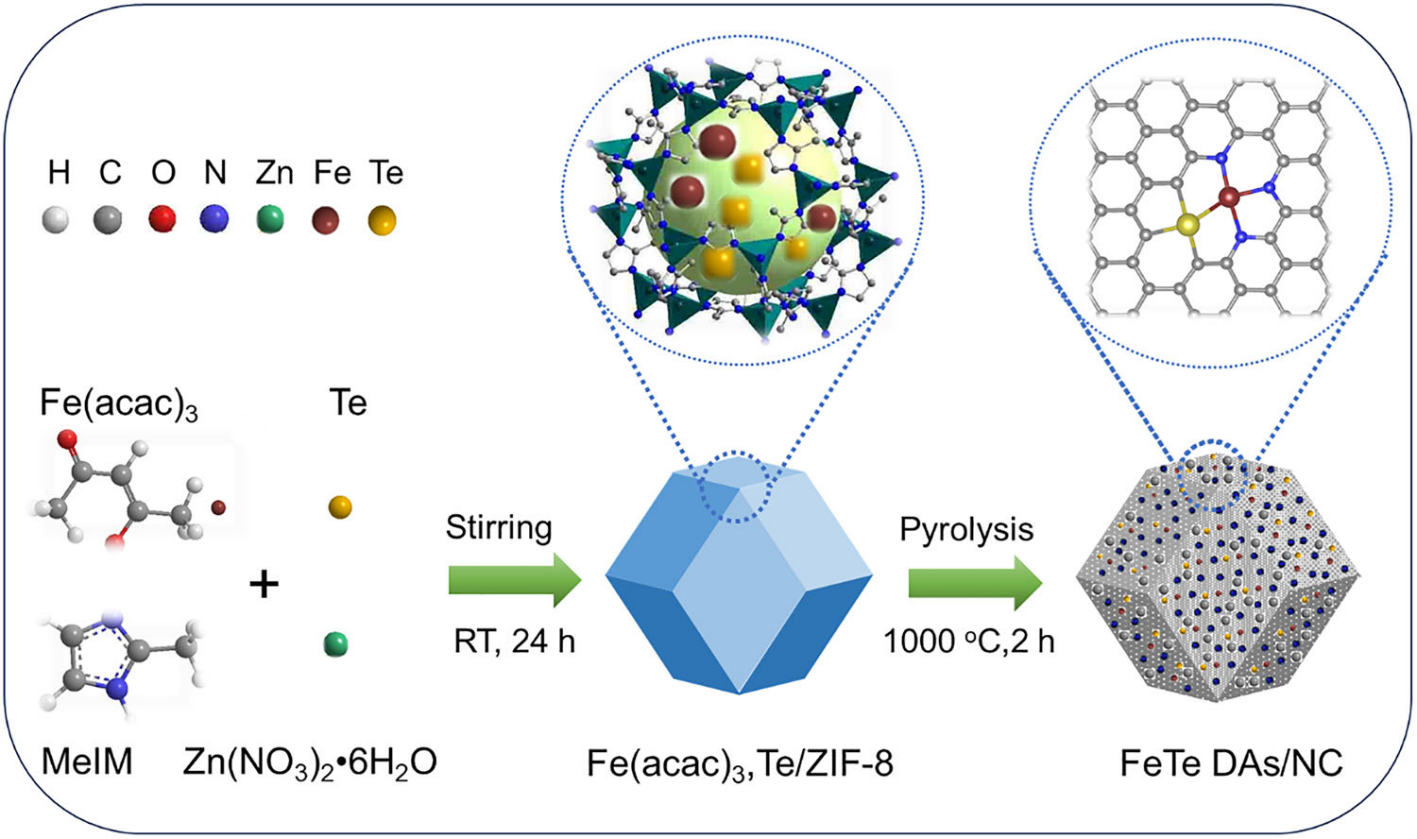
Schematic illustration of the synthesis process of FeTe DAs/NC.

The FeTe DAs/NC catalyst was synthesized via a coordination‐assisted assembly and pyrolysis strategy (Figure 1). Fe(acac)_3_ and Te powder were co‐introduced during the formation of ZIF‐8 by adding them together with Zn^2+^ and 2‐methylimidazole (MeIM) into a solvent mixture of DMF, methanol and ethylene glycol. For comparison, Fe SAs/NC and Te SAs/NC were prepared in the absence of Te or Fe precursors, respectively. The actual loadings of Fe and Te were determined via inductively coupled plasma mass spectroscopy (ICP‐MS), as listed in Table S1. Powder x‐ray diffraction (PXRD) patterns (Figure S1) confirm that the ZIF‐8 framework is retained in all precursors, although Te/ZIF‐8 shows slightly reduced crystallinity due to the physical incorporation of Te. The Brunauer–Emmett–Teller (BET) surface areas of the ZIF‐8 precursors range from 1284 to 1432 m^2^ g^−1^ (Figure S2), with a consistent pore size distribution centered at ∼1.1 nm (Figure S3), indicating that neither Fe nor Te incorporation disrupted the framework structure. Thermogravimetric analysis (Figure S4) revealed a major weight loss at around 450°C due to framework decomposition, which guided the selection of a pyrolysis temperature of 1000°C for Zn removal and catalyst formation [26]. The post‐pyrolysis PXRD patterns of FeTe DAs/NC, Fe SAs/NC, and Te SAs/NC displayed broad peaks near 26° and 44°, assignable to graphitic carbon, without any detectable crystalline Fe or Te species (Figure S5), suggesting atomic dispersion or ultrasmall clusters. Raman spectra revealed I_D_/I_G_ values of 2.457, 2.187, and 3.035 for FeTe DAs/NC, Fe SAs/NC, and Te SAs/NC, respectively (Figure S6), indicating a decrease in graphitization with increasing Te content. A distinct Te─C stretching vibration at 471 cm^−1^ further confirmed Te incorporation. Nitrogen sorption analysis showed that FeTe DAs/NC, Fe SAs/NC, and Te SAs/NC retained abundant microporosity with comparable pore size distributions (Figures S7 and S8), facilitating mass transport and catalytic site accessibility during PMS activation.

**FIGURE 2:**
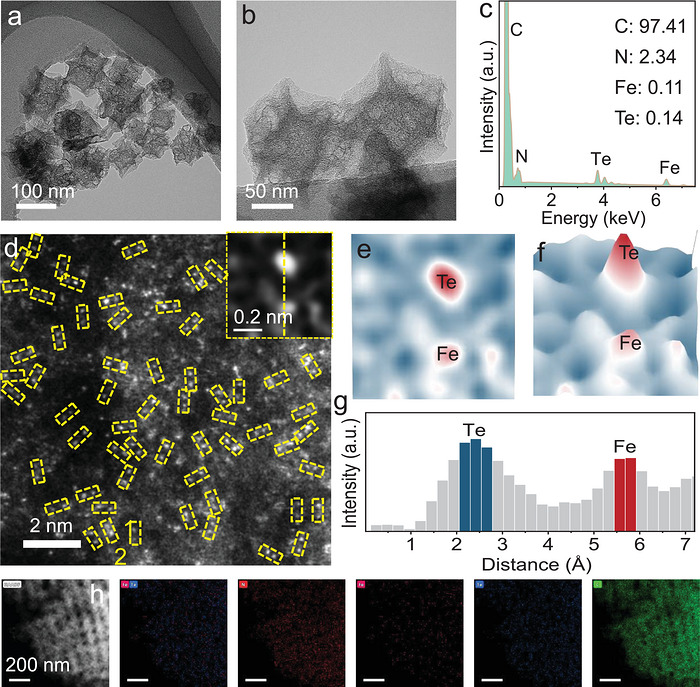
Morphology characterization of FeTe DAs/NC. (a) HRTEM image and (b) the magnified HR‐TEM image. (c) EDS elemental composition. (d) AC‐HAADF–STEM image (inset: magnified dual‐atom pair image). (e and f) Corresponding 2D and 3D topographic images from region 2 highlighted in (d). (g) Line‐scanning intensity profiles obtained from the inset in (d). (h) EDS elemental mapping images.

Scanning electron microscopy (SEM, Figure S9) and high‐resolution transmission electron microscopy (HR‐TEM, Figure 2a) images reveal that the FeTe DAs/NC exhibit a rougher surface after pyrolysis, which facilitates the exposure of the active site. The enlarged HR‐TEM image (Figure 2b) shows no discernible nanoparticles or clusters corresponding to Fe or Te species, suggesting atomic‐level dispersion. Elemental analysis (Figure 2c) confirms the presence of C, N, Fe and Te, with no detectable Zn, indicating complete volatilization during pyrolysis. Aberration‐corrected high‐angle annular dark‐field scanning transmission electron microscopy (AC‐HAADF‐STEM, Figure 2d) further reveals atomically dispersed Fe and Te atoms, evident from the high‐density bright dots highlighted in the rectangular region. The contrast differences in 2D and 3D topological projections (Figure 2e,f) show higher intensity for Te atoms compared to Fe, consistent with their relative atomic numbers. The atomic pairs, with an interatomic distance of ∼2.8–3.0 Å (Figure 2g), confirm the formation of asymmetric Fe–Te dual‐atom sites. Energy‐dispersive spectroscopy (EDS) mapping (Figure 2h) displays a homogeneous distribution of C, N, Fe, and Te throughout the porous carbon matrix, with interconnected ∼20 nm pores providing channels for mass transport. Comparative SEM images (Figures S10 and S11) show that Te SAs/NC exhibit a rougher surface than Fe SAs/NC. Additional HAADF–STEM and EDS mappings (Figures S12 and S13) corroborate the atomically dispersed nature and homogeneous elemental distribution of Fe and Te within the N‐doped carbon framework.

**FIGURE 3:**
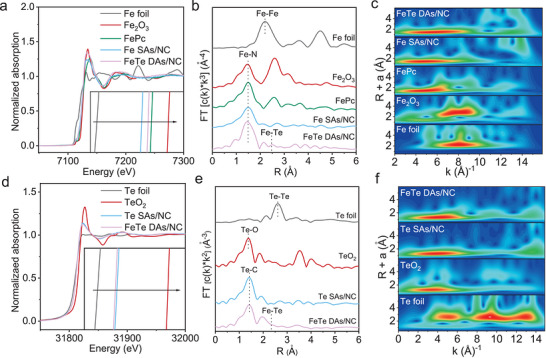
(a) XANES spectra of Fe K‐edge, (b) FTs of κ^3^‐weighted Fe K‐edge EXAFS data, and (c) WTs for the κ^3^‐weighted Fe K‐edge EXAFS signals. (d) XANES spectra of Te K‐edge, (e) FTs of κ^3^‐weighted Te K‐edge EXAFS data, and (f) WTs for the κ^3^‐weighted Te K‐edge EXAFS signals.

The X‐ray photoelectron spectrum was used to analyze the chemical state. For the N 1s spectra of FeTe DAs/NC and Fe SAs/NC (Figures S14 and S15), four peaks at 398.2, 399.0, 400.7 and 401.9 eV can be assigned to the pyridinic N, Fe–N, pyrrolic N and graphitic N, respectively. The content of graphitic N decreases and no peak attributable to Fe–N is observed in Te SAs/NC (Figure S16). In the Te 3d spectra, the Te 3d_5/2_ peaks for Te SAs/NC appear at 574.1 and 576.2 eV (Figures S17 and S18), positioned between those of Te^0^ (573.1 eV) and Te^4+^ in TeO_2_ (576.3 eV), indicating a positively charged state. FeTe DAs/NC also displays positively charged Te species, with a negative shift of ∼0.3 eV, suggesting the formation of *p–d* hybridization and electron redistribution toward Te. To further elucidate the oxidation states and coordination environments, X‐ray absorption near‐edge structure (XANES) and extended X‐ray absorption fine structure (EXAFS) measurements were performed (Figures 3 and S19–S22). At the Fe K‐edge (Figure 3a), the absorption edge of FeTe DAs/NC lies between those of Fe foil and FePc, indicating an intermediate oxidation state. Linear combination fitting (LCF) analysis shows that the average oxidation state of Fe in FeTe DAs/NC increases to +2.15 compared with +1.87 of Fe SAs/NC. The Fourier‐transformed (FT) EXAFS spectra at the Fe K‐edge (Figure 3b) show dominant Fe─N coordination at ∼1.45 Å with a coordination number of ∼4.0 and ∼3.0  for FeTe DAs/NC and Fe SAs/NC, respectively, consistent with wavelet transform (WT) results (Figure 3c), confirming the atomically dispersed nature of Fe. Additionally, a weak peak at ∼2.5 Å in FeTe DAs/NC cannot be attributed to Fe‐N or Fe‐Fe interactions and is instead assigned to Fe–Te coordination, with a coordination number of ∼0.8 (Tables S2 and S3), consistent with the atomic distances observed in the STEM images. At the Te K‐edge, both Te SAs/NC and FeTe DAs/NC show absorption edges between those of metallic Te and TeO_2_ (Figure 3d), with the average oxidation state of Te in FeTe DAs/NC decreasing to +0.93 from +1.13 in Te SAs/NC. Combined with the change in Fe oxidation state, it further confirms electron redistribution between Fe *d* orbitals and Te *p* orbitals in asymmetric Fe–Te dual‐atom pairs, forming *p–d* hybridization, in which the electron density is shifted toward Te due to its higher electronegativity. The FT‐EXAFS spectra (Figure 3e) exhibit a distinct peak at ∼1.35 Å, attributed to Te─C coordination (Figure 3f), confirming the atomic dispersion of Te within the carbon matrix.

### 2.2 Atomic Asymmetry Steers PMS Activation for Pollutant Degradation

**FIGURE 4:**
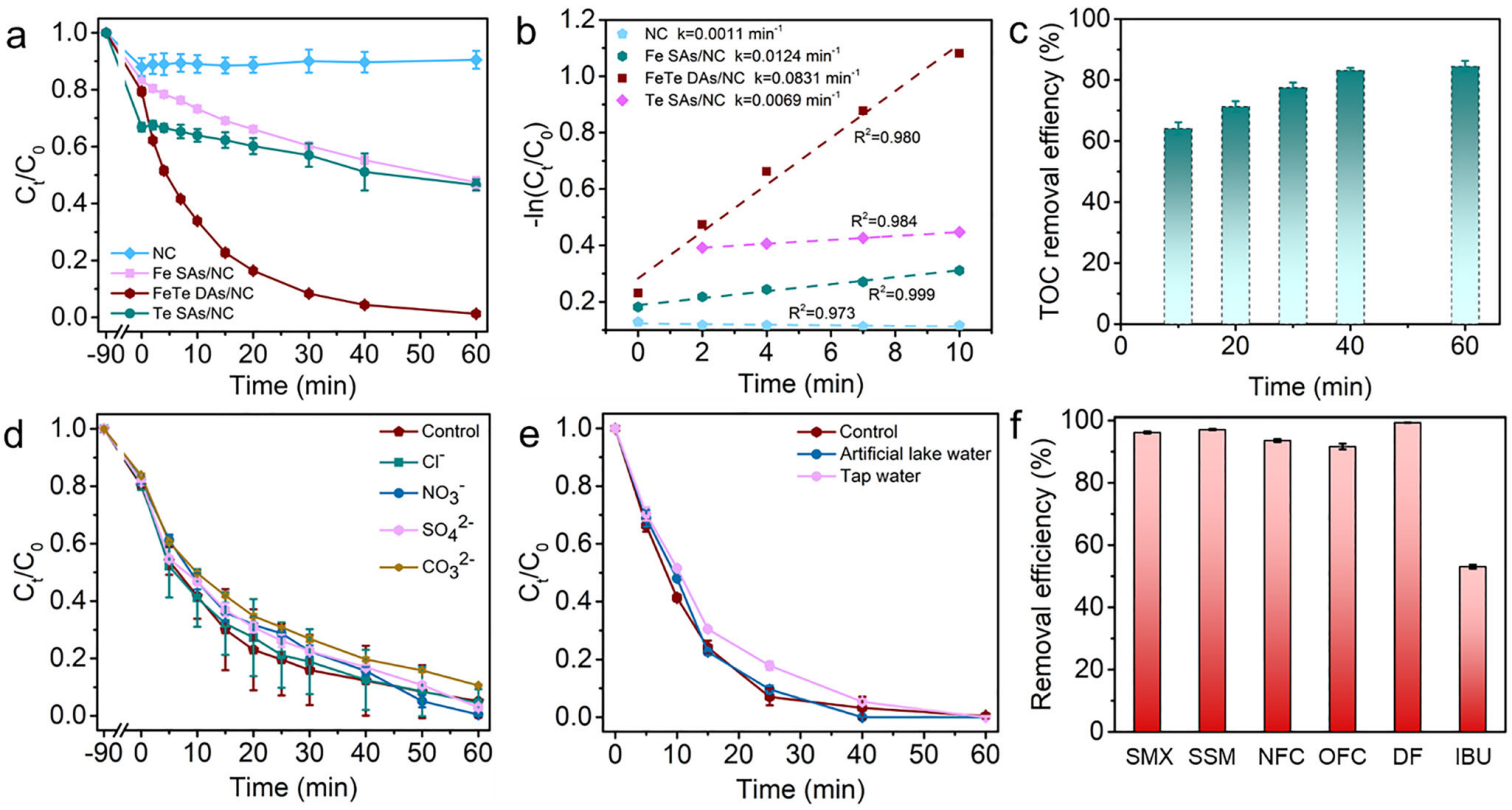
(a) CBZ degradation when Fe SAs/NC, FeTe DAs/NC, Te SAs/NC and NC were used as catalysts and (b) the corresponding kinetic constants. (c) TOC removal in the process of CBZ degradation, (d) Effect of different ions on CBZ degradation, (e) degradation of CBZ in environmental water samples, and (f) different Pollutants of degradation removal in the FeTe DAs/NC system with PMS. (Experimental conditions: 100 mL reaction solution, 10 mg L^−1^ pollutants, 2 mM PMS, 100 mg L^−1^ catalyst, 6.0 pH).

The catalytic activities of FeTe DAs/NC, Fe SAs/NC, Te SAs/NC, and NC were evaluated using carbamazepine (CBZ) as a model pollutant. In the absence of PMS, CBZ removal was attributed solely to adsorption, with Te SAs/NC showing the highest adsorption capacity (Figure 4a). Upon PMS addition, FeTe DAs/NC exhibited significantly enhanced activity, achieving complete CBZ degradation within 60 min. In contrast, both Fe SAs/NC and Te SAs/NC in the presence of PMS showed limited activity, with CBZ removal efficiencies of ∼50%, while NC displayed a negligible catalytic effect despite 90% removal due to pure adsorption. Kinetic analysis based on pseudo‐first‐order fitting (Figure 4b) revealed that the apparent rate constant for FeTe DAs/NC was 6.5 and 11.8 times higher than those of Fe SAs/NC and Te SAs/NC. Total organic carbon (TOC) analysis further confirmed the mineralization performance, with TOC removal reaching 60% within 10 min and stabilizing at ∼80% (Figure 4c), underscoring the superior oxidative degradation capability of FeTe DAs/NC. To elucidate the degradation process, intermediate products were detected by LC‐MS (Figure S23a). Two main pathways were proposed: pathway 1 (Transformation product 1, TP1‐TP3) and pathway 2 (TP1 to TP4‐TP6) (Figure S23b) [27, 28]. In Path 1, TP1 (detected at 10 min) and TP2 (detected at 20 min) were generated via hydroxylation of CBZ, indicative of ^•^OH radical attack. TP2 underwent sequential ring‐opening and intramolecular cyclization to afford a dialdehyde intermediate, followed by cleavage of acetyl and aldehyde moieties to yield TP3 (detected at 10 min). In Path 2, cleavage of the C─N bond in TP1 gave rise to TP4 (detected at 10 min), whose aldehyde group was further eliminated to form TP5 (detected at 10 min), which was then oxidized to TP6 within the initial 10 min of degradation, thus contributing to TOC reduction. Finally, these intermediates were continuously attacked by ROS to generate smaller molecular fragments: TP7 (dominant at 20 min), TP8 (detected at 10 min), and TP9 (dominant at 20 min). These species were ultimately mineralized into H_2_O, CO_2_, NH_3,_ and other byproducts, accounting for the high TOC removal observed within the first 10 min. To assess the influence of coexisting anions, four common species (Cl^−1^ 30 mg L^−1^, NO_3_^−1^ mg L^−1^, SO_4_^2−^ 50 mg L^−1^, CO_3_^2−^ 50 mg L^−1^) were introduced (Figure 4d). Among them, only CO_3_^2−^ led to reduced degradation efficiency, likely due to pH variation, whereas the others showed negligible effects. Furthermore, CBZ degradation was evaluated in practical water matrices, including artificial lake water and tap water (Figure 4e), where the system maintained the high catalytic performance, demonstrating its robustness in complex environments. Reusability tests confirmed that FeTe DAs/NC retained ∼100% degradation efficiency over five cycles (Figure S24), with minimal Fe and Te leaching (∼100 and ∼20 µg L^−1^, respectively), well below safety thresholds. The kinetic constant remained stable at 0.08 min^−1^, confirming the long‐term durability of the catalyst, which was further confirmed by XRD and Raman spectra (Figures S25–S27). Finally, to further assess its versatility, six representative pharmaceuticals (sulfamethoxazole (SMX), sulfisoxazole (SSM), norfloxacin (NFC), ofloxacin (OFC), diclofenac (DF), and ibuprofen (IBU)) were tested (Figure 4f). Except for IBU with ∼52% removal efficiency and 0.0094 min^−1^ degradation rate constant, all other pollutants were degraded with efficiencies exceeding 90% within 60 min and higher degradation rate constants (Table S4), further indicating the strong catalytic performance of the FeTe DAs/NC‐PMS system.

### 2.3 Mechanism of Catalytic Degradation

**FIGURE 5:**
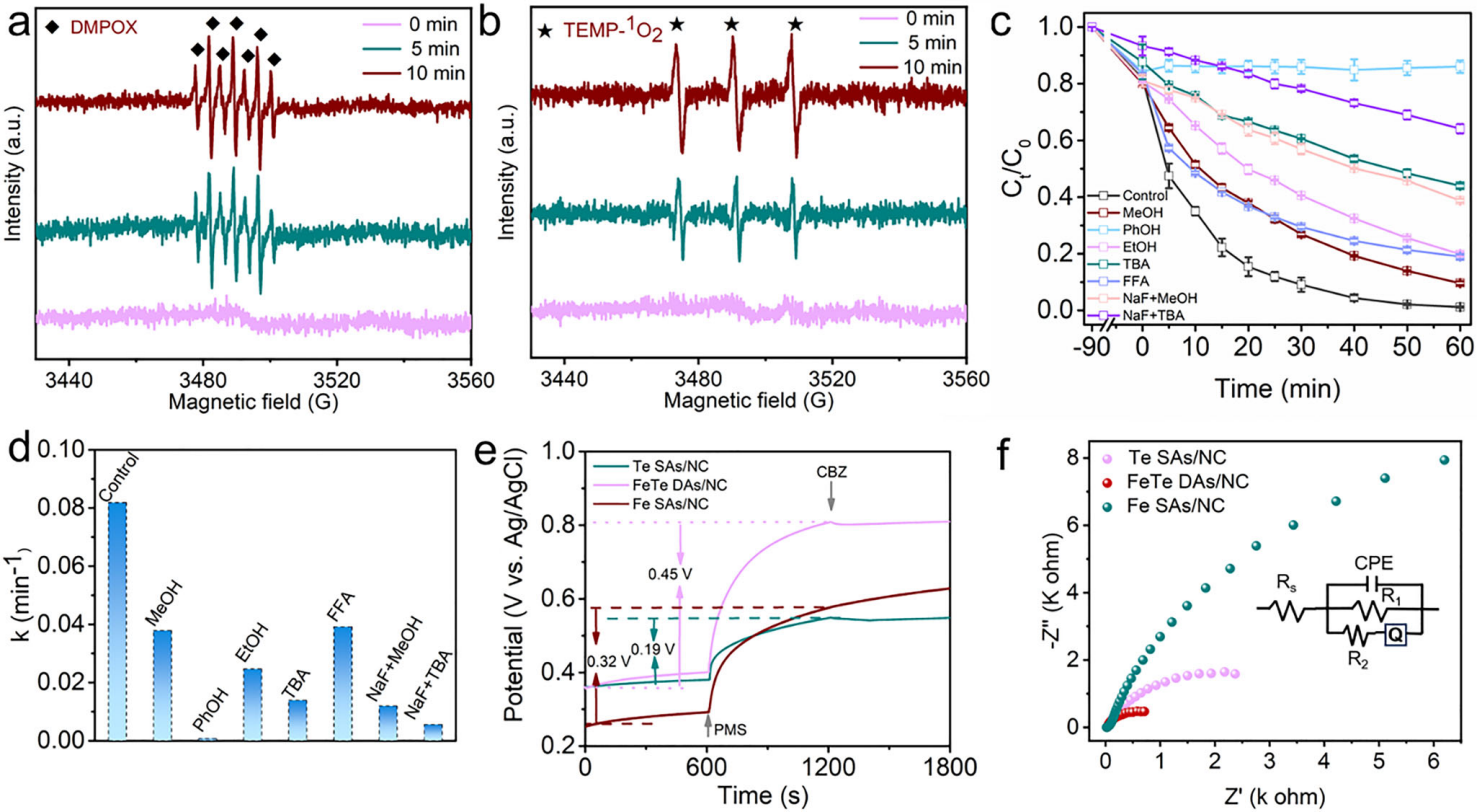
(a) DMPO and (b) TEMP spin‐trapping EPR spectra of FeTe DAs/NC system with PMS. (c) Quenching experiments for the degradation of CBZ in the FeTe DAs/NC‐PMS system and (d) the corresponding kinetic constants. (e) Open‐circuit potential (OCP) curves monitoring upon sequential injection of PMS and SMX, and (f) EIS spectra of Fe SAs/NC, FeTeDAs/NC, and Te SAs/NC (Inset: equivalent circuit diagram). (Experimental conditions: 100 mL reaction solution, 10 mg L^−1^ pollutants, 2 mM PMS, 100 mg L^−1^ catalyst, 6.0 pH).

Electron paramagnetic resonance (EPR) spectroscopy was employed to identify reactive oxygen species (ROS) in the FeTe DAs/NC‐PMS system. Compared with Fe SAs/NC and Te SAs/NC, a characteristic seven‐line EPR signal of 5,5‐dimethyl‐1‐pyrrolidone‐N‐oxyl (DMPOX) was observed only in FeTe DAs/NC (Figures 5a and S28 and S29) [11, 29]. Since DMPOX typically originates from the oxidation of DMPO by an intense burst of ^•^OH, high‐valent metal species, or singlet oxygen (^1^O_2_), control experiments were conducted to clarify the origin [30, 31]. When water was replaced with methanol (MeOH), the characteristic peaks of DMPOX disappeared completely (Figures S30–S32), indicating that radical pathways (^•^OH or SO_4_^•−^) are involved [30]. Further EPR measurements confirmed the generation of ^1^O_2_ in the FeTe DAs/NC–PMS system at both 5 and 10 min (Figure 5b), whereas the signals in Fe SAs/NC and Te SAs/NC appeared significantly weaker (Figures S33 and S34), indicating a synergistic effect in the dual‐site system.

To evaluate the contributions of reactive oxygen species toward CBZ degradation, quenching experiments using furfuryl alcohol (FFA, 5 mM) were performed [32]. Even upon efficient quenching of ^1^O_2_, the removal efficiency of CBZ remained at 80% (*k* = 0.0392 min^−1^), indicating that ^1^O_2_ plays only a minor role in both DMPO oxidation and CBZ degradation (Figure 5c,d). Similarly, quenching tests with dimethylsulfoxide (DMSO) (5 mM) ruled out the involvement of high‐valent iron species in the oxidation of DMPO (Figure S35). In contrast, radical (^•^OH and SO_4_^•−^) scavengers MeOH (500 mM) and EtOH (500 mM) showed moderate inhibition on CBZ degradation (90% removal efficiency and k = 0.0379 min^−1^, 80% removal efficiency and k = 0.0247 min^−1^), while tertiary butanol (TBA, 500 mM), a selective ^•^OH scavenger [33], exhibited the strongest suppression (50% removal efficiency, *k* = 0.0139 min^−1^), implicating ^•^OH as the primary ROS to oxidize the DMPO into DMPOX in EPR results. The partial inhibition by TBA indicates surface‐bound ^•^OH species, rather than free radicals in solutions. However, TBA did not completely prohibit the CBA degradation, suggesting that ^•^OH is primarily surface‐bound on the catalyst [9, 34]. Phenol (PhOH, 5 mM) is commonly used to probe the role of surface‐confined species. Upon its addition to the FeTe DAs/NC‐PMS system, the removal efficiency is just 12%, and the kinetic constant is close to 0, showing that the surface active species is the dominant ROS responsible for CBZ degradation [10, 34]. Notably, compared with the Fe SAs/NC and Te SAs/NC systems (Figures S36 and S37), FeTe DAs/NC exhibited a significantly greater suppression of CBZ removal upon TBA addition, further confirming the dominant contribution of surface‐confined ^•^OH in the dual‐atom catalyst system. Upon addition of fluoride ions (F^−^_,_ 5 mM), which promote the desorption of surface‐bound ^•^OH via hydrogen bonding [34, 35, 36], the inhibitory effects of MeOH (500 mM) and TBA (500 mM) were further enhanced (Figure 5c,d). This observation indicates that surface‐confined ^•^OH was gradually released into the solution and participated in pollutant degradation. These findings are consistent with the observed TOC removal (∼80% within 60 min), highlighting the sustained activity and effective utilization of surface‐bound ROS.

To reveal electron transfer behavior, open‐circuit potential (OCP) measurements were conducted (Figure 5e). Upon PMS addition, the OCP of the FeTe DAs/NC system increased by 0.45 V, higher than those of Fe SAs/NC (0.32 V) and Te SAs/NC (0.19 V), indicating stronger electron‐transfer interactions with PMS in the asymmetric dual‐site system. No significant OCP change was observed after the addition of CBZ, suggesting limited direct electron transfer between CBZ and the catalyst. Chronoamperometry measurements (Figure S38) showed an increased current upon PMS addition, whereas no noticeable change occurred after CBZ introduction [37], further excluding a direct electron transfer pathway. These observations suggest the formation of a metastable PMS/FeTe DAs/NC surface complex as the active platform for ROS generation [3]. Electrochemical impedance spectroscopy (EIS) reveals that FeTe DAs/NC has the lowest interface resistance (R_s_, R_1_, and R_2_) among all catalysts (Figure 5f; Table S5). The CPE elements from the equivalent circuit diagram also show the higher capacitance with greater active surface area, underscoring the role of asymmetric dual‐atom sites in enhancing electron transfer kinetics during ROS activation.

### 2.4 DFT Calculations

**FIGURE 6:**
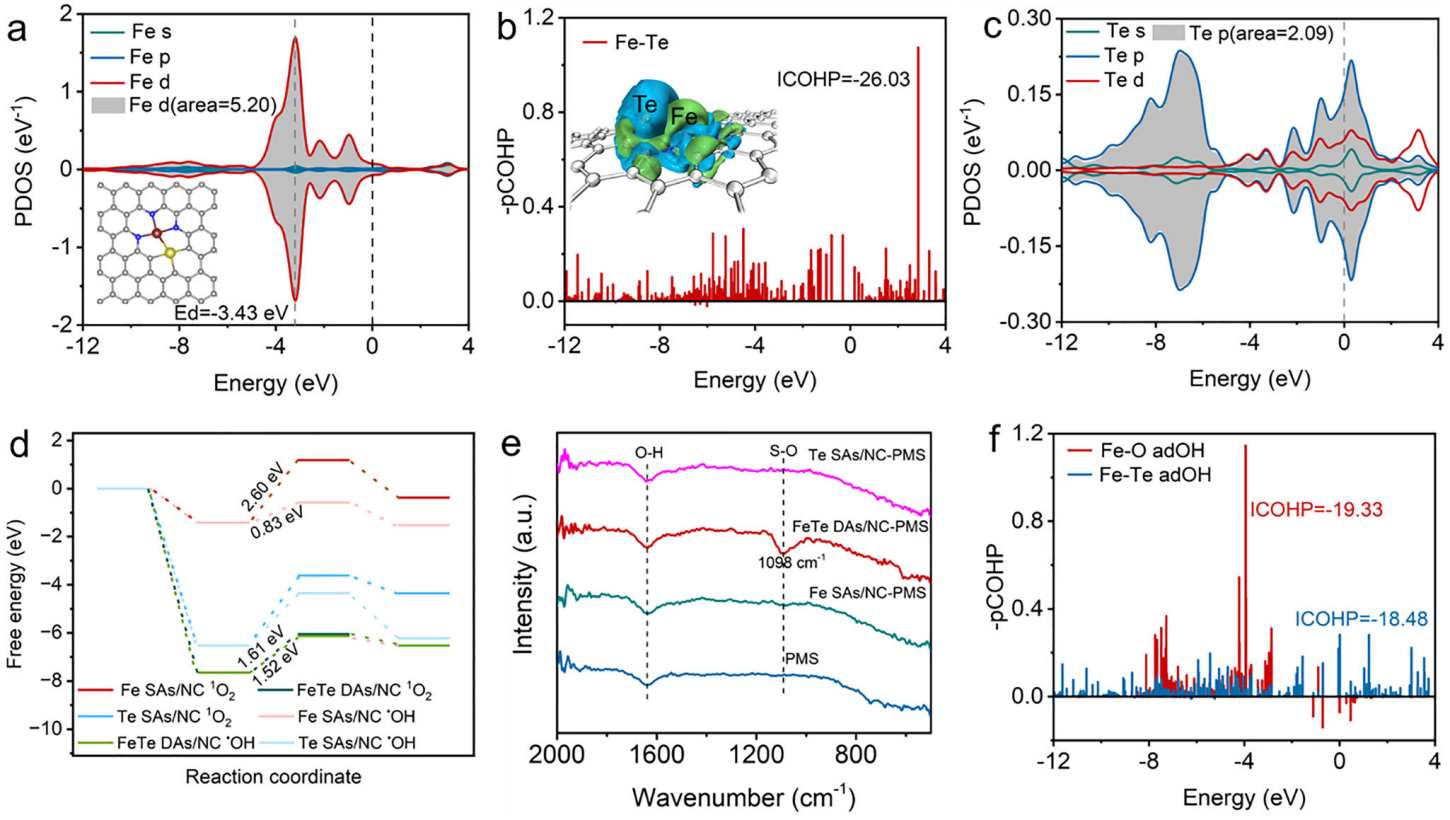
(a) Partial density of states for Fe of FeTe DAs/NC (inset: atomic configuration of FeTe‐N_3_C_3_). (b) COHP data of Fe *d* orbitals and Te *p* orbitals on the FeTe DAs/NC (Inset: differential charge distribution diagram). (c) Partial density of states for Te of FeTe DAs/NC. (d) Gibbs free energy of PMS and intermediates absorbed on Fe‐N_4_, FeTe‐N_3_C_3_, and Te‐C_4_. (e) In situ ATR‐FTIR spectra of PMS, Fe SAs/NC‐PMS, FeTe DAs/NC‐PMS, and Te SAs/NC‐PMS. (f) pCOHP analysis of the Fe atom and O/Te atom of *OH species on the FeTe DAs/NC.

Density functional theory (DFT) calculations were conducted to elucidate the role of *p–d* orbital coupling in modulating PMS activation. As shown in the projected density of states (PDOS, Figures 6a and S39), the Fe *d*‐band center in FeTe DAs/NC (−3.43 eV) was slightly lower than that in Fe SAs/NC (−3.39 eV), suggesting a more favorable adsorption energy for key intermediates. Moreover, the Fe *d*‐orbital density increased from 4.57 to 5.20, indicating strong Fe–Te electronic coupling. The orbital contributions around the Fermi level were dominated by Fe 3d and Te 5p states, and the negative integrated crystal orbital Hamilton population (ICOHP) confirms the formation of covalent Fe–Te interactions (Figure 6b). Significant orbital overlaps between Fe 3d and Te 5p was observed below the Fermi level (∼5 eV), corroborated by a notably negative ICOHP value of −26.03, further evidencing *p–* *d* orbital hybridization [23]. To directly visualize the electronic coupling between Fe and Te atoms, differential charge density calculations were performed (Inset of Figure 6b). Pronounced charge redistribution is observed at the Fe–Te interface, with electron depletion regions (cyan) concentrated around Te atoms and electron accumulation regions (lime) localized on Fe atoms. This distinct charge separation clearly reveals significant electron transfer from Te to Fe. Quantitative Bader charge analysis further indicates that Te bears a positive charge of +0.576 e, whereas Fe carries a charge of +0.037 e. Such an electronic configuration demonstrates that Te incorporation tailors the electronic structure of Fe sites, stabilizing them in a slightly electron‐deficient metastable state. Notably, the synergistic effect of these two positively charged centers generates a strongly electrophilic environment, which greatly facilitates PMS activation and strengthens electrostatic attraction toward negatively charged HSO_5_^−^, thereby promoting subsequent S─O bond cleavage. Analysis of the Te *p*‐orbital PDOS (Figures 6c and S40) showed a substantial increase in FeTe DAs/NC (2.09) compared to Te SAs/NC (0.16), confirming substantial electron donation from Fe to Te and strong orbital mixing between Fe and Te. This electron delocalization around Fe enhances PMS adsorption, as reflected by the more negative PMS adsorption energy on FeTe DAs/NC relative to Fe SAs/NC and Te SAs/NC (Figure 6d). This was further validated by in situ ATR‐FTIR, where a prominent S─O vibrational band at 1098 cm^−1^ appeared in the FeTe DAs/NC‐PMS system (Figure 6e) [10], confirming effective PMS adsorption and activation.

For the rate‐determining step (RDS) of generating ^•^OH/^1^O_2_, except for the pathway of generating ^•^OH in the Fe SAs/NC‐PMS system with a lower 0.83 eV energy barrier, the pathway of generating ^•^OH/^1^O_2_ in the FeTe DAs/NC‐PMS system presents the lower energy barriers than Fe SAs/NC‐PMS and Te SAs/NC‐PMS systems, with 1.61 and 1.52 eV, respectively. These findings suggest a synergistic Fe–Te interaction that facilitates PMS activation and ROS production. However, the desorption of ^•^OH/^1^O_2_ from FeTe DAs/NC was thermodynamically less favorable than in the other two systems, implying a higher probability of surface‐bound ROS retention. With respect to surface hydroxyl radicals, desorption via Fe─O bond cleavage requires overcoming a thermodynamic barrier of −2.6 eV. This arises because the Fe atom strengthens its binding to the oxygen species by partially reducing its orbital overlap with Te. Consequently, when hydroxyl radicals are present on the surface, the bonding contribution in the Fe–O COHP overwhelmingly dominates (Figure 6f), yielding an ICOHP of −19.33 eV. Meanwhile, the presence of surface radicals decreases the Fe–Te ICOHP to −18.48 eV, highlighting that the *d–p* orbital overlap between the Fe and Te atoms plays a critical role in stabilizing surface oxygen‐containing radicals [24]. These results are consistent with the experimental observation that surface‐confined ^•^OH is the dominant reactive species. Overall, the *p–d* orbital hybridization induced by Te incorporation facilitates electron delocalization at the Fe site, leading to enhanced PMS adsorption and ROS activation, ultimately contributing to the formation and retention of surface‐bound ^•^OH.

## 3 Conclusion

In summary, we have developed an asymmetric Fe–Te pairs catalyst (FeTe DAs/NC) featuring strong *p–d* orbital hybridization, which enables efficient PMS activation and selective generation of surface‐bound ^•^OH for the robust degradation of emerging micropollutants. Compared with single‐atom analogs, FeTe DAs/NC delivers markedly improved catalytic activity and mineralization efficiency, achieving complete carbamazepine degradation within 60 min while maintaining high tolerance toward complex aqueous environments. Mechanistic studies reveal that the incorporation of Te induces *p–d* orbital coupling and electron delocalization at the Fe center, enhancing PMS adsorption, lowering activation barriers, and suppressing ^•^OH desorption to prolong ROS surface retention. This work not only advances the fundamental understanding of dual‐atom catalysis in PMS‐based advanced oxidation processes (AOPs) but also provides a rational design principle for constructing next‐generation environmental catalysts via asymmetric metal–metalloid pairing.

## Author Contributions

Y.S., T.Z., and J.‐M.L. conceived and designed the research. X.L. and Y.S. synthesized the samples and performed the characterizations. Y.S. supported the XAS experiments and data analysis. X.L. and S.W. carried out the measurements. C.W. and X.L. performed and analyzed the DFT calculations. Y.S. drafted the manuscript. X.L, Y.S., T.Z., M.Z., and J.‐M.L. discussed, and all authors reviewed and edited the manuscript.

## Conflicts of Interest

The authors declare no conflict of interest.

## Supporting information




**Supporting File 1**: anie72466‐sup‐0001‐SuppMat.docx

## Data Availability

The data that supports the findings of this study are available in the Supporting Information of this article

## References

[1] X. Wang , Y. Wang , Z. Zhang , et al., “Effect, Fate and Remediation of Pharmaceuticals and Personal Care Products (PPCPs) During Anaerobic Sludge Treatment: A Review,” Environmental Science & Technology 58 (2024): 19095–19114, 10.1021/acs.est.4c06760. 39428634

[2] I. C. Iakovides , V. G. Beretsou , A. Christou , et al., “Impact of the Wastewater Treatment Technology and Storage on Micropollutant Profiles During Reclaimed Water Irrigation: A Wide‐scope HRMS Screening in a Water‐Soil‐Lettuce‐Leachate System,” Water Research 279 (2025): 123319, 10.1016/j.watres.2025.123319. 40132301

[3] Q. Zhou , C. Song , P. Wang , Z. Zhao , Y. Li , and S. Zhan , “Generating Dual‐Active Species by Triple‐Atom Sites Through Peroxymonosulfate Activation for Treating Micropollutants in Complex Water,” Proceedings National Academy of Science USA 120 (2023): e2300085120, 10.1073/pnas.2300085120. PMC10068799 36952382

[4] J. Lee , U. von Gunten , and J.‐H. Kim , “Persulfate‐Based Advanced Oxidation: Critical Assessment of Opportunities and Roadblocks,” Environmental Science & Technology 54 (2020): 3064–3081, 10.1021/acs.est.9b07082. 32062964

[5] H. Dong , Q. Xu , L. Lian , et al., “Degradation of Organic Contaminants in the Fe(II)/Peroxymonosulfate Process Under Acidic Conditions: The Overlooked Rapid Oxidation Stage,” Environmental Science & Technology 55 (2021): 15390–15399, 10.1021/acs.est.1c04563. 34730346

[6] C. Gao , Y. Su , X. Quan , et al., “Electronic Modulation of Iron‐Bearing Heterogeneous Catalysts to Accelerate Fe(III)/Fe(II) Redox Cycle for Highly Efficient Fenton‐Like Catalysis,” Applied Catalysis B: Environment and Energy 276 (2020): 119016, 10.1016/j.apcatb.2020.119016.

[7] Q.‐Y. Wu , Z.‐W. Yang , Z.‐W. Wang , and W.‐L. Wang , “Oxygen Doping of Cobalt‐Single‐Atom Coordination Enhances Peroxymonosulfate Activation and High‐Valent Cobalt–Oxo Species Formation,” Proceedings National Academy of Science USA 120 (2023): e2219923120, 10.1073/pnas.2219923120. PMC10120063 37040400

[8] Z.‐H. Xie , C.‐S. He , H.‐Y. Zhou , et al., “Effects of Molecular Structure on Organic Contaminants′ Degradation Efficiency and Dominant ROS in the Advanced Oxidation Process With Multiple ROS,” Environmental Science & Technology 56 (2022): 8784–8795, 10.1021/acs.est.2c00464. 35584301

[9] Y. Feng , P.‐H. Lee , D. Wu , and K. Shih , “Surface‐Bound Sulfate Radical‐dominated Degradation of 1,4‐dioxane by Alumina‐supported Palladium (Pd/Al_2_O_3_ ) Catalyzed Peroxymonosulfate,” Water Research 120 (2017): 12–21, 10.1016/j.watres.2017.04.070. 28478290

[10] C. Chen , M. Yan , Y. Li , et al., “Single‐Atom co Sites Confined in Layered Double Hydroxide for Selective Generation of Surface‐bound Radicals via Peroxymonosulfate Activation,” Applied Catalysis B: Environment and Energy 340 (2024): 123218, 10.1016/j.apcatb.2023.123218.

[11] Y. Xiong , H. Li , C. Liu , et al., “Single‐Atom Fe Catalysts for Fenton‐like Reactions: Roles of Different N Species,” Advanced Materials 34 (2022): 2110653, 10.1002/adma.202110653. 35263466

[12] X. Yu , H. Liu , Y. Huang , et al., “A Green Edge‐hosted Zinc Single‐Site Heterogeneous Catalyst for Superior Fenton‐Like Activity,” Proceedings National Academy of Science USA 120 (2023): e2221228120, 10.1073/pnas.2221228120. PMC10450848 37590415

[13] X. Zhou , M. K. Ke , G. X. Huang , et al., “Identification of Fenton‐Like Active Cu Sites by Heteroatom Modulation of Electronic Density,” Proceedings National Academy of Science USA 119 (2022): e2119492119, 10.1073/pnas.2119492119. PMC8872710 35165185

[14] Y. Wang , T. Lan , L. Han , et al., “Non‐Precious Metal Catalysts With Gradient Oxidative Dual Sites Boost Bimolecular Activation for Catalytic Oxidation Reactions,” Angewandte Chemie International Edition 64 (2025): e202506018, 10.1002/anie.202506018. 40202179 PMC12171384

[15] J. Sun , L. Tao , C. Ye , et al., “MOF‐Derived Ru_1_Zr_1_ /Co Dual‐Atomic‐Site Catalyst With Promoted Performance for Fischer–Tropsch Synthesis,” Journal of the American Chemical Society 145 (2023): 7113–7122, 10.1021/jacs.2c09168. 36951270

[16] S. Wang , X. Hou , Y. Li , C. Zhou , P. Zhang , and C. Hu , “From Single‐Atom to Dual‐Atom: A Universal Principle for the Rational Design of Heterogeneous Fenton‐Like Catalysts,” Environmental Science & Technology 59 (2025): 8822–8833, 10.1021/acs.est.4c13826. 40261206

[17] F. Wang , Y. Gao , H. Fu , et al., “Almost 100 % Electron Transfer Regime Over Fe−Co Dual‐atom Catalyst Toward Pollutants Removal: Regulation of Peroxymonosulfate Adsorption Mode,” Applied Catalysis B: Environment and Energy 339 (2023): 123178, 10.1016/j.apcatb.2023.123178.

[18] Z. Zhao , M. Hu , T. Nie , et al., “Improved Electronic Structure From Spin‐State Reconstruction of a Heteronuclear Fe–Co Diatomic Pair to Boost the Fenton‐Like Reaction,” Environmental Science & Technology 57 (2023): 4556–4567, 10.1021/acs.est.2c09336. 36894515

[19] Y. Huang , Y. Zhao , D. Song , et al., “Overlooked Synergistic Effect Induced by Fe‐Mo Dual‐atom Catalyst in Water Remediation: Enhanced Electron Management and Efficient Pollutants Pre‐activation,” Applied Catalysis B: Environment and Energy 374 (2025): 125377, 10.1016/j.apcatb.2025.125377.

[20] Z. Chen , F. An , Y. Zhang , Z. Liang , W. Liu , and M. Xing , “Single‐Atom Mo–Co Catalyst With Low Biotoxicity for Sustainable Degradation of High‐ionization‐potential Organic Pollutants,” Proceedings National Academy of Science USA 120 (2023): e2305933120, 10.1073/pnas.2305933120. PMC10629517 37428912

[21] J. Jiang , S. Liu , B. Zhao , et al., “Angstrom Confinement‐Triggered Adaptive Spin State Transition of CoMn Dual Single Atoms for Efficient Singlet Oxygen Generation,” Advanced Materials 37 (2025): 2417834, 10.1002/adma.202417834. 39901371

[22] Y. Chen , H. Zhang , Y. Li , W.‐W. Li , G.‐P. Sheng , and Y. Wang , “Coordination Anions Dimensionality‐Engineered Dual‐Atom Catalysts for Enhanced Fenton‐Like Reactions: 3D Coordination Induced Spin‐State Transition,” ACS Nano 19 (2025): 14187–14199, 10.1021/acsnano.5c00567. 40183629

[23] X. Wang , N. Zhang , S. Guo , et al., “p‐d Orbital Hybridization Induced by Asymmetrical FeSn Dual Atom Sites Promotes the Oxygen Reduction Reaction,” Journal of the American Chemical Society 146 (2024): 21357–21366, 10.1021/jacs.4c03576. 39051140

[24] B. Peng , H. She , Z. Wei , et al., “Sulfur‐Doping Tunes p‐d Orbital Coupling Over Asymmetric Zn‐Sn Dual‐atom for Boosting CO_2_ Electroreduction to Formate,” Nature Communications 16 (2025): 2217, 10.1038/s41467-025-57573-4. PMC11882884 40044667

[25] Y. Liu , J. Li , Z. Lv , et al., “Efficient Proton‐Exchange Membrane Fuel Cell Performance of Atomic Fe Sites via p–d Hybridization With Al Dopants,” Journal of the American Chemical Society 146 (2024): 12636–12644, 10.1021/jacs.4c01598. 38676645

[26] X. Xie , C. He , B. Li , et al., “Performance Enhancement and Degradation Mechanism Identification of a Single‐atom Co–N–C Catalyst for Proton Exchange Membrane Fuel Cells,” Nature Catalysis 3 (2020): 1044–1054, 10.1038/s41929-020-00546-1.

[27] Y. Wang , T. Zhou , D. Chen , et al., “Fenton‐Like Catalysis by MnO_2_ Membrane Reactor With Oxygen Vacancies for Carbamazepine Degradation,” Applied Catalysis B: Environment and Energy 353 (2024): 124106, 10.1016/j.apcatb.2024.124106.

[28] X. Li , H. Zhang , J. Liu , et al., “Revealing the Overlooked Catalytic Ability of γ‐Al_2_O_3_: Efficient Activation of Peroxymonosulfate for Enhanced Water Treatment,” Environmental Science & Technology 58 (2024): 22466–22476, 10.1021/acs.est.4c08834. 39627152

[29] B. Wang , C. Cheng , M. Jin , et al., “A Site Distance Effect Induced by Reactant Molecule Matchup in Single‐Atom Catalysts for Fenton‐like Reactions,” Angewandte Chemie International Edition 61 (2022): e202207268, 10.1002/anie.202207268. 35719008

[30] F. Mo , C. Song , Q. Zhou , et al., “The Optimized Fenton‐Like Activity of Fe Single‐atom Sites by Fe Atomic Clusters–Mediated Electronic Configuration Modulation,” Proceedings National Academy of Science USA 120 (2023): e2300281120, 10.1073/pnas.2300281120. PMC10104488 37011202

[31] J.‐H. Wu and H.‐Q. Yu , “How Do Metal Oxides Mislead Spin‐Trapping Electron Paramagnetic Resonance Analysis?,” Environmental Science & Technology Letters 11 (2024): 370–375, 10.1021/acs.estlett.4c00155.

[32] W. Tang , H. Zhang , X. Yang , et al., “Ru Single Atom Catalyst With Dual Reaction Sites for Efficient Fenton‐Like Degradation of Organic Contaminants,” Applied Catalysis B: Environment and Energy 320 (2023): 121952, 10.1016/j.apcatb.2022.121952.

[33] J. Jiang , Z. Zhao , J. Gao , et al., “Nitrogen Vacancy‐Modulated Peroxymonosulfate Nonradical Activation for Organic Contaminant Removal via High‐Valent Cobalt‐Oxo Species,” Environmental Science & Technology 56 (2022): 5611–5619, 10.1021/acs.est.2c01913. 35442647

[34] N. Chen , G. Fang , C. Zhu , et al., “Surface‐Bound Radical Control Rapid Organic Contaminant Degradation Through Peroxymonosulfate Activation by Reduced Fe‐bearing Smectite Clays,” Journal of Hazardous Materials 389 (2020): 121819, 10.1016/j.jhazmat.2019.121819. 31848100

[35] G. Fang , Y. Deng , M. Huang , D. D. Dionysiou , C. Liu , and D. Zhou , “A Mechanistic Understanding of Hydrogen Peroxide Decomposition by Vanadium Minerals for Diethyl Phthalate Degradation,” Environmental Science & Technology 52 (2018): 2178–2185, 10.1021/acs.est.7b05303. 29376648

[36] X. Zhang , H. Xiao , Q. Xu , et al., “Characterization of Phthalides in Ligusticum Chuanxiong by Liquid Chromatographic‐Atmospheric Pressure Chemical Ionization‐Mass Spectrometry,” Journal of Chromatographic Science 41(2003): 428–433, 10.1093/chromsci/41.8.428. 14558936

[37] Z.‐Y. Guo , R. Sun , Z. Huang , et al., “Crystallinity Engineering for Overcoming the Activity–Stability Tradeoff of Spinel Oxide in Fenton‐Like Catalysis,” Proceedings National Academy of Science USA 120 (2023): e2220608120, 10.1073/pnas.2220608120. PMC10104503 37018199

