# Supplementary material for: Asymmetric Fe–Te Pairs Enhance Peroxymonosulfate Activation via Surface‐Bound Hydroxyl Radicals Pathways
Source: Angew Chem Int Ed Engl. 2026 May 2;65(25):e3343591. doi: 10.1002/anie.3343591 (PMC13266939; doi:10.1002/anie.3343591)
Supplement: Supplementary file 1 — Supporting File 1: anie72466‐sup‐0001‐SuppMat.docx [file ANIE-65-e3343591-s001.docx]

**Supplementary Information**

**Asymmetric Fe–Te Pairs Enhance Peroxymonosulfate Activation via Surface-Bound Hydroxyl Radicals Pathways**

Xuheng Li^‡^,^1^ Chunli Wang^‡^,^4^ Yuntong Sun,^*3^ Sheng Wang,^5^ Min Zheng,^4^ Tierui Zhang,^*2^ Jong-Min Lee^*3,6,7^

^1^ School of Chemistry and Chemical Engineering, Xi'an University of Architecture and Technology, Xi'an, Shaanxi, 710055 China

^2^ Key Laboratory of Photochemical Conversion and Optoelectronic Materials, Technical Institute of Physics and Chemistry, Chinese Academy of Sciences, Beijing 100190, China

^3^ School of Chemistry, Chemical Engineering and Biotechnology, Nanyang Technological University, 62 Nanyang Drive, Singapore, 637459 Singapore

^4^ School of Chemical Engineering, Faculty of Sciences, Engineering and Technology, The University of Adelaide, Adelaide, South Australia, Australia

^5^ State Key Laboratory of Water Pollution Control and Green Resource Recycling, Shanghai Institute of Pollution Control and Ecological Security, School of Environmental Science and Engineering, Tongji University, Shanghai 200092, China.

^6^ Department of Energy Science and Engineering, Daegu Gyeongbuk Institute of Science and Technology (DGIST), Daegu 42988, Republic of Korea

^7^ Energy Science and Engineering Research Center, Daegu Gyeongbuk Institute of Science and Technology (DGIST), Daegu 42988, Republic of Korea

‡ The authors contributed equally to this work

^*^Corresponding authors

E-mails: yuntong.sun@ntu.edu.sg; tierui@mail.ipc.ac.cn; lee@dgist.ac.kr

**I. Experimental Section**

**1. Materials and reagents**

Carbamazepine (CBZ), sulfamethoxazole (SMX), sulfaclozine (SSM), norfloxacin (NFC), ofloxacin (OFC), diclofenac (DF), ibuprofen (IBU), methanol and humic acid (HA) are purchased from Sigma-Aldrich Co. Ltd. (99%). Phenol (C_6_H_6_O, ≥99%), Sodium sulfate (Na_2_SO_4_, ≥99%), Acetonitrile (CH_3_CN, ≥99.9%), Ammonium acetate (C_2_H_7_NO_2_, ≥99.9%), Sodium chloride (NaCl, ≥99.9%), Oxalic acid (C_2_H_2_O_4_, ≥99.9%), Sodium hydroxide (NaOH, ≥99.9%), Hydrochloric acid (HCl, ≥99.9%), Ethanol (CH_3_CH_2_OH, ≥99.5%), Methanol (CH_3_OH, 99.5%), tert-Butanol (C_4_H_10_O, ≥99.5%), Sodium bicarbonate (NaHCO_3_, ≥99.8%), Sodium nitrate (NaNO_3_, 99.9%), Potassium phosphate monobasic (KH_2_PO_4_, ≥99.9%), Dimethyl sulfoxide (C_2_H_6_SO, ≥99.9%), Hydroxylammonium chloride (H_3_NO·HCl, 99%), Potassium iodide (KI, ≥99.0%) were obtained from Aladdin Biochemical Technology Co., Ltd. (Shanghai, China). 5,5-Dimethyl-1-pyrroline N-oxide (C_6_H_11_NO, 97%), L-Histidine (C_6_H_9_N_3_O_2_, 99%), Methyl phenyl sulfoxide (C_7_H_8_OS, 98%), Methyl phenyl sulfone (C_7_H_8_O_2_S, 98%), Potassium monopersulfate triple salt (K_5_H_3_S_4_O_18_, 42-46% KHSO_5_ basis), 2,2,6,6-Tetramethyl-4-piperidinol (C_9_H_19_NO, 99%) were purchased from Shanghai Macklin Biochemical Co., Ltd. All chemicals were used without further purification. Ultrapure water (18.2 MΩ cm) was used in the experiment.

**2. Material synthesis**

**Synthesis of** **FeTe DAs/NC.** FeTe dual-atom catalysts supported on nitrogen-doped carbon (FeTe DAs/NC) were synthesized via a ZIF-8−derived strategy. In a typical procedure, 1.19 g Zn(NO_3_)_2_·6H_2_O, 0.0706 g Fe(acac)_3_ and 0.0255 g Te powder were added into 36 mL DMF, 12 mL methanol and 12 mL ethylene glycol under stirring for 15 min. Separately, 1.314 g of 2-H-MeIM was dissolved in 12 mL DMF and 8 mL methanol. Then, two solutions were mixed under stirring and further stirring at room temperature for 24 h, and the resulting precipitate was collected by centrifugation and washed thoroughly with methanol three times. The reuslted Fe(acac)_3_/Te@ZIF-8 powders were transferred to ceramic boats and placed in a tube furnace. The furnace was heated to 1,000 °C at a heating rate of 2 °C min^−1^, then held at 1000 °C for 2 h under an Ar atmosphere before being naturally cooled to room temperature. The obtained materials were directly used without additional treatment.

**Synthesis of Fe SAs/NC, Te SAs/NC, and NC.** The Fe SAs/NC, Te SAs/NC and nitrogen-doped carbon (NC) were prepared using the same procedure as FeTe DAs/NC, except that only Fe(acac)_3_ (0.100 g), only Te powder (0.075 g), or neither Fe(acac)_3_ nor Te was introduced during the precursor synthesis step, respectively..

**3. Physical Characterization**

Morphological features were observed on field emission scanning electron microscope (FESEM, JEOL 7800F), transmission electron microscope (TEM), high resolution transmission electron microscope (HRTEM, Titan G2 60-300, a spherical and chromatic aberration imaging corrector). Energy-dispersive X-ray spectroscopy (EDS) and element mapping were acquired on STEM (150~230Mx 200 KV). Crystal structure was examined by X-ray diffractometer (XRD, D8 Advance, Bruker, Karlsruhe, Germany 9 kw, 40 kV, 40 mA, λ=1.5418 Å) with Cu-Kα radiation. Chemical state was analyzed by X-ray photoelectron spectroscopy (XPS, Thermo ESCALAB 250XI between 0 and 1400 eV). Organic functional group were recorded by Fourier transform infrared spectroscopy (FTIR spectra, Thermofisher NICOLETIS 10 FTIR spectrometer) and Raman spectroscopy using the excitation wavelength of 532 nm (HORIBA JOBIN YVON, Te RAMAN-1024x256-OPEN-SYN). The adsorption capacity for N_2_ was collected by the temperature-programmed desorption of N_2_ (N_2_-TPD, Autochem 2920 facility). The composition of metal in catalysts were determined by inductively coupled plasma mass spectrometer (ICP-MS, NexIONTM 350D). EPR spectroscopy was performed using a Bruker EMX Micro spectrometer equipped with a Bruker ER4123- D dielectric resonator which were operated at room temperature.

**4. Electrochemical characterizations**

All the electrochemical tests were conducted in a three- electrode cell connected to an electrochemical workstation (CHI 760E). Pt wire and Ag/AgCl electrode were used as the counter and reference electrodes, respectively. The working electrode was the catalyst-modified carbon paper (1×3 cm^2^). The Na_2_SO_4_ (50 mM, pH = 6.0) solution was used as an electrolyte unless otherwise specified. The catalyst ink was prepared by sufficiently sonicating the mixture of the catalyst (6 mg), isopropyl alcohol (2.22 mL), Nafion solution (0.06 mL), and DI H_2_O (1.44 mL). The carbon paper was then modified with the catalyst ink (10 µL) to achieve the desired catalyst loading amount (0.1 mg cm^−2^). The open-circuit potential and current of the system with the catalyst-loaded electrode and PMS were monitored by chronopotentiometry and chronoamperometry analysis. PMS and CBZ were added at given intervals to final concentrations of 50 mg L^−1^ and 10 mg L^−1^, respectively.

**5. Evaluation of the Catalytic Performance**

The Fenton-like system was assessed in a 100 mL reactor containing 50 mL model pollutant (10 mg L^−1^) solution maintained at 25 °C using a water bath. In a typical experiment, 5 mg of catalyst was added to the above solution with an initial solution pH of 7.2. The oxidation reaction was then initiated by adding a certain amount of PMS aqueous solution to achieve the desired concentration (2 mM). Not otherwise specified, the experiments were performed at an initial pH of 7.2, and they cannot be performed at higher pH (pH > 8.0) as PMS could be activated by alkali. At predetermined time intervals, 0.5 mL of the reaction solution was withdrawn and immediately filtered by a 0.45 μm PTFE membrane into a high-performance liquid chromatography (HPLC) vial containing 20 μL of sodium thiosulfate solution (0.1 M) to terminate the catalytic reaction. Unless otherwise specified, all kinetic experiments were carried out in deionized water produced by the Lab water system (Dura Elit 10F). The concentration of CBZ, SMX, SSM, NFC, OFC, DF, and IBU was analyzed by HPLC (Agilent, 1260Infinity) with a C18 column at λ_DAD_ = 285 nm, 270 nm, 272 nm, 278 nm, 278 nm, 276 nm, and 210 nm, respectively. The mobile phase was a mixture of methanol/ Milli-Q water dissolved with ammonium acetate and formic acid. The volume ratio is 70:30, 30:70, 40:60, 35:65, 35:65, 80:20, and 75:25, respectively. Intermediate products during CBZ degradation were detected using high-performance liquid chromatography coupled with quadrupole time-of-flight mass spectrometry (HPLC-QTOF-MS, 1260–6520, Agilent).

**6.** **DFT Calculation Details**

We performed all calculations of the first principle employing the CP2K^[1]^ program package. All the atoms were described by the DZVP-MOLOPT-SR-GTH basis sets.^[2]^ In the mixed Gaussian-plane-wave scheme (GPW), the atomic core electrons are described by Goedecker−Teter−Hutter pseudopotentials.^[3]^ The Perdew-Burke-Ernzerhof (PBE) functional with D3^[4]^ correction was adopted for describing the electronic structure,^[5, 6]^ and a cutoff of 450 Ry for the auxiliary plane-wave basis set was used. A graphene-p(6×6) supercell was built as the basic model for materials. At least 20 Å of vacuum was set to separate the successive slabs in all calculations. Geometry optimization was performed until all the forces in the atoms were smaller than 0.25 eV nm^–1^. For the free energy of adsorbate in the liquid phase of a specific concentration, we calculated the value under equilibrium partial pressure equivalently.^[7, 8]^ When adsorbed masses are adsorbed on a slab, the contribution of simple harmonic vibrations is considered when calculating their free energy.

II. Supplementary Results

**Figure S1.** Powder X-ray diffraction pattern of Fe, Te/ZIF-8, Fe/ZIF-8, Te/ZIF-8.

**Figure S2.** N_2_ adsorption−desorption isotherms (at 77 K) of ZIF-8, Te/ZIF-8, Fe, Te/ZIF-8, and Fe/ZIF-8.

**Figure S3.** Pore distribution of ZIF-8, Te/ZIF-8, Fe,Te/ZIF-8, and Fe/ZIF-8.

**Figure S4.** Thermogravimetric curves of ZIF-8, Te/ZIF-8, Fe,Te/ZIF-8, and Fe/ZIF-8 under N_2_ flow.

**Figure S5.** Powder X-ray diffraction pattern of FeTe DAs/NC, Fe SAs/NC, Te SAs/NC, and NC.


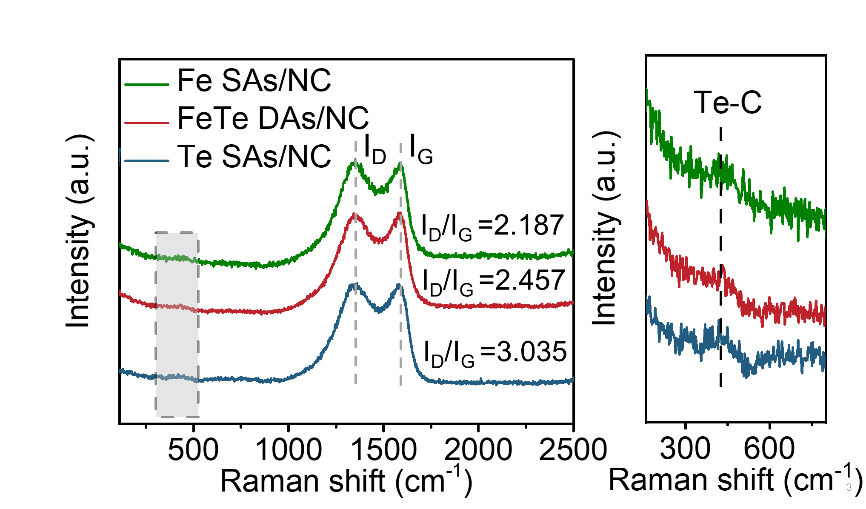


**Figure S6.** Raman spectra of FeTe DAs/NC, Fe SAs/NC, Te SAs/NC.





**Figure S7.** N_2_ adsorption−desorption isotherms (at 77 K) of Te SAs/NC, FeTe DAs/NC, Fe SAs/NC, and NC.





**Figure S8.** Pore distribution of Te SAs/NC, FeTe DAs/NC, Fe SAs/NC, and NC.


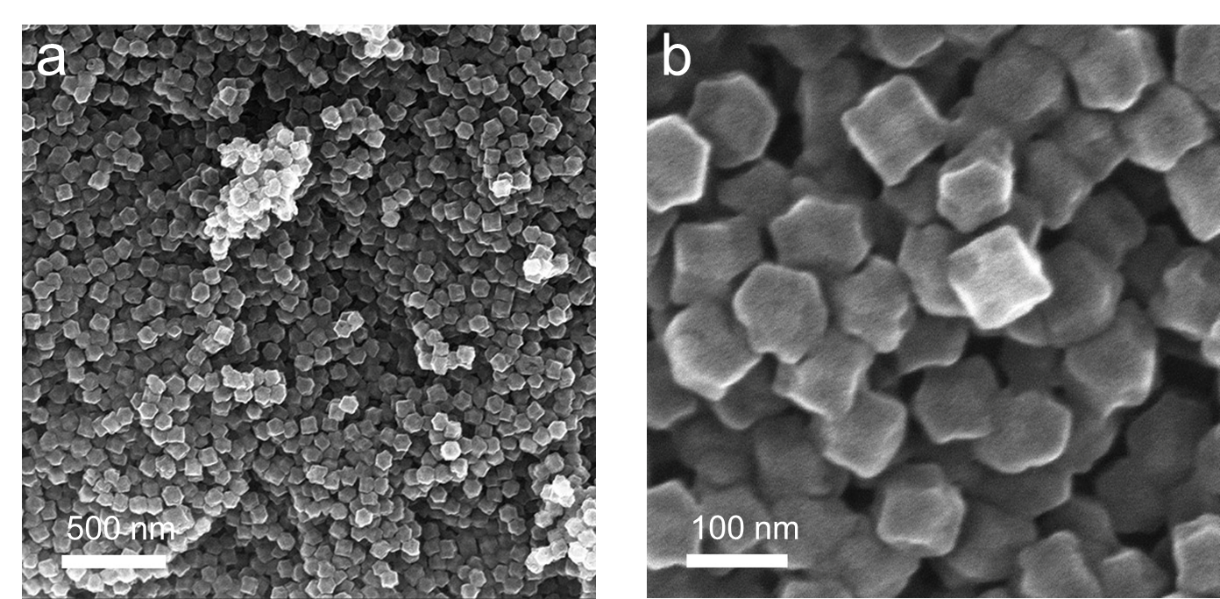


**Figure S9.** (a, b) SEM images of FeTe DAs/NC.


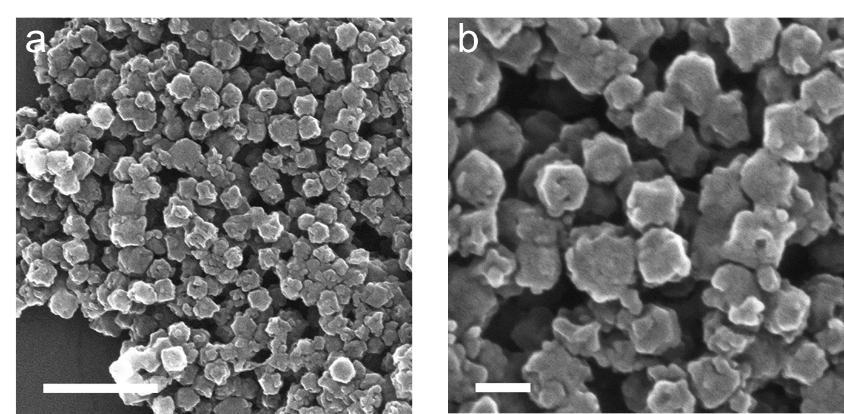


**Figure S10.** (a, b) SEM images of Te SAs/NC.


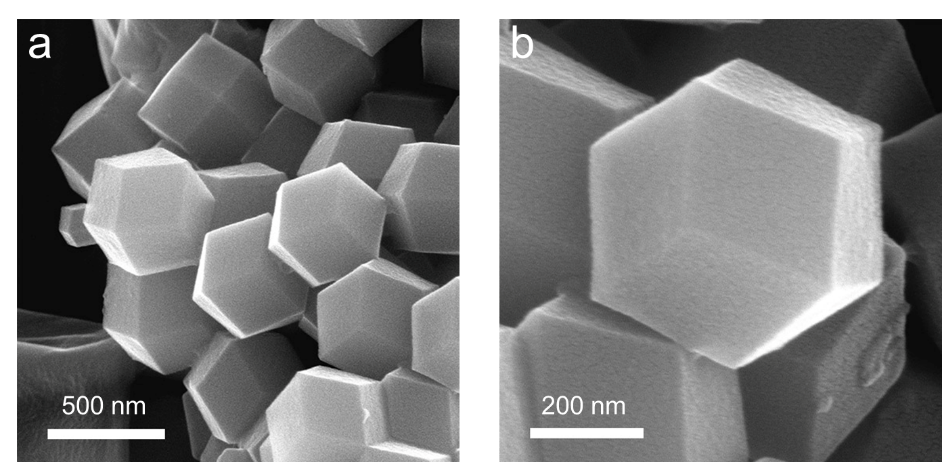


**Figure S11.** SEM images of Fe SAs/NC.


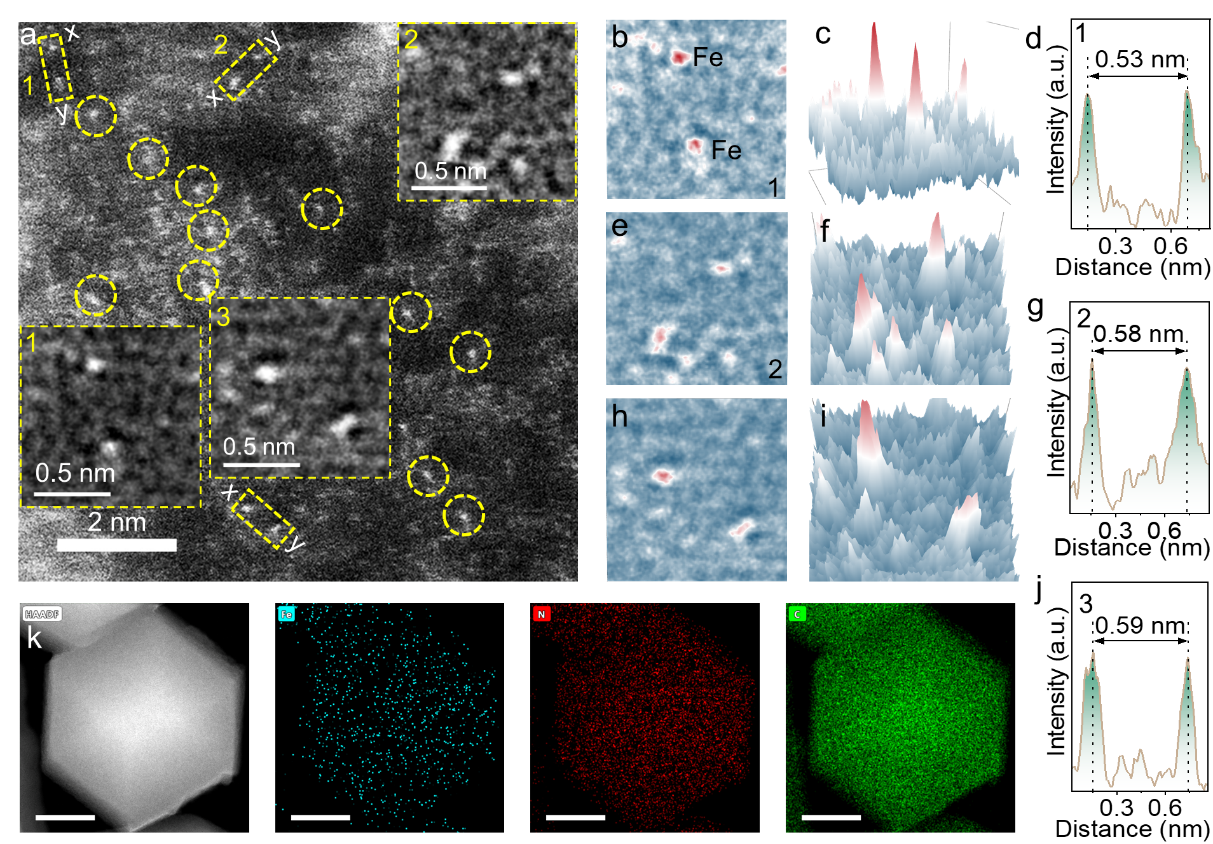


**Figure S12.** Morphology characterization of Fe SAs/NC. (a) AC-HAADF-STEM image. (b-c, e-f, and h-i) Corresponding 2D and 3D topographic images from the square region 1, 2, 3 highlighted in (a). (d, g, and j) Line-scanning intensity profiles obtained from the rectangular region 1–3 in (a). (k) EDS elemental composition.


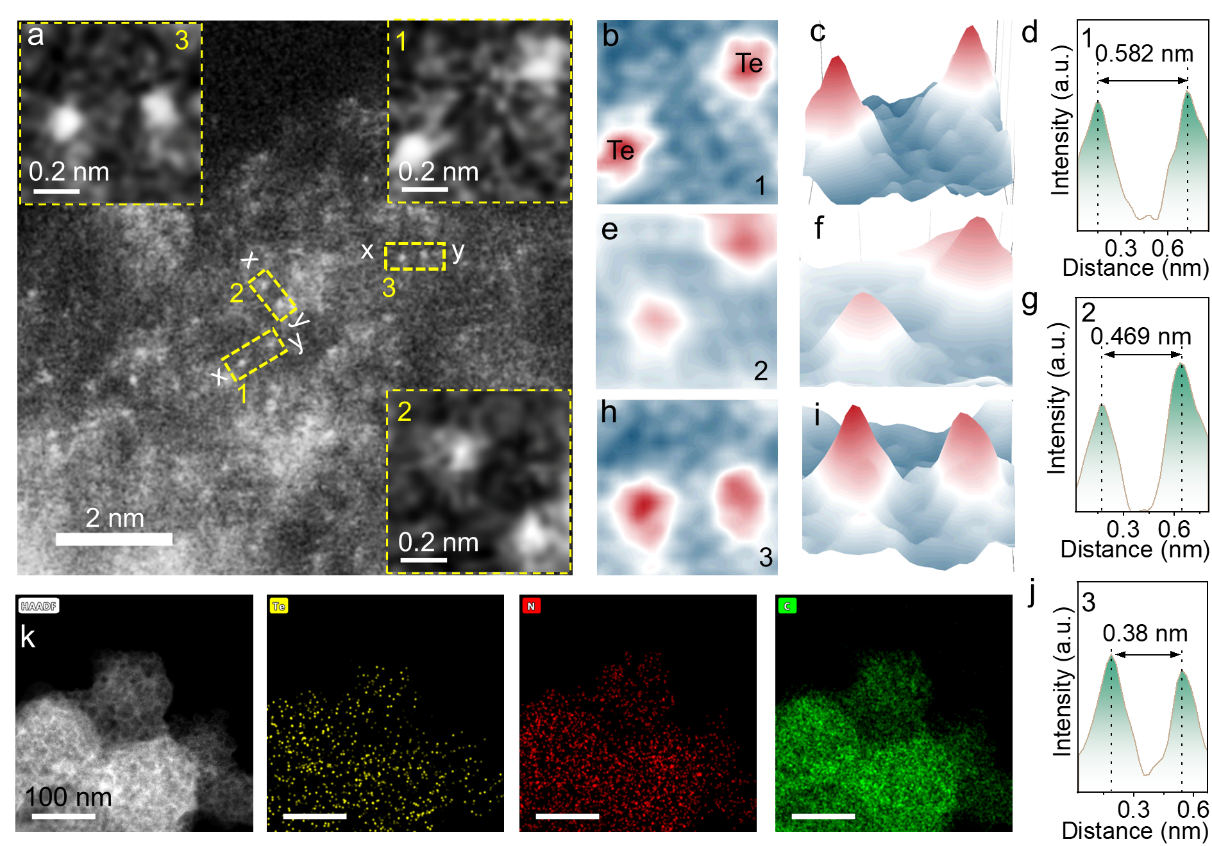


**Figure S13.** Morphology characterization of Te SAs/NC. (a) AC-HAADF-STEM image. (b-c, e-f, and h-i) Corresponding 2D and 3D topographic images from the square region 1, 2, 3 highlighted in (a). (d, g, and j) Line-scanning intensity profiles obtained from the rectangular region 1–3 in (a). (k) EDS elemental composition.





**Figure S14.** XPS spectra for N 1s in FeTe DAs/NC.





**Figure S15.** XPS spectra for N 1s spectra in Fe SAs/NC.





**Figure S16.** XPS spectra for N 1s in Te SAs/NC.





**Figure S17.** XPS spectra for Te 3d spectra in FeTe DAs/NC.





**Figure S18.** XPS spectra for Te 3d spectra in Te SAs/NC.


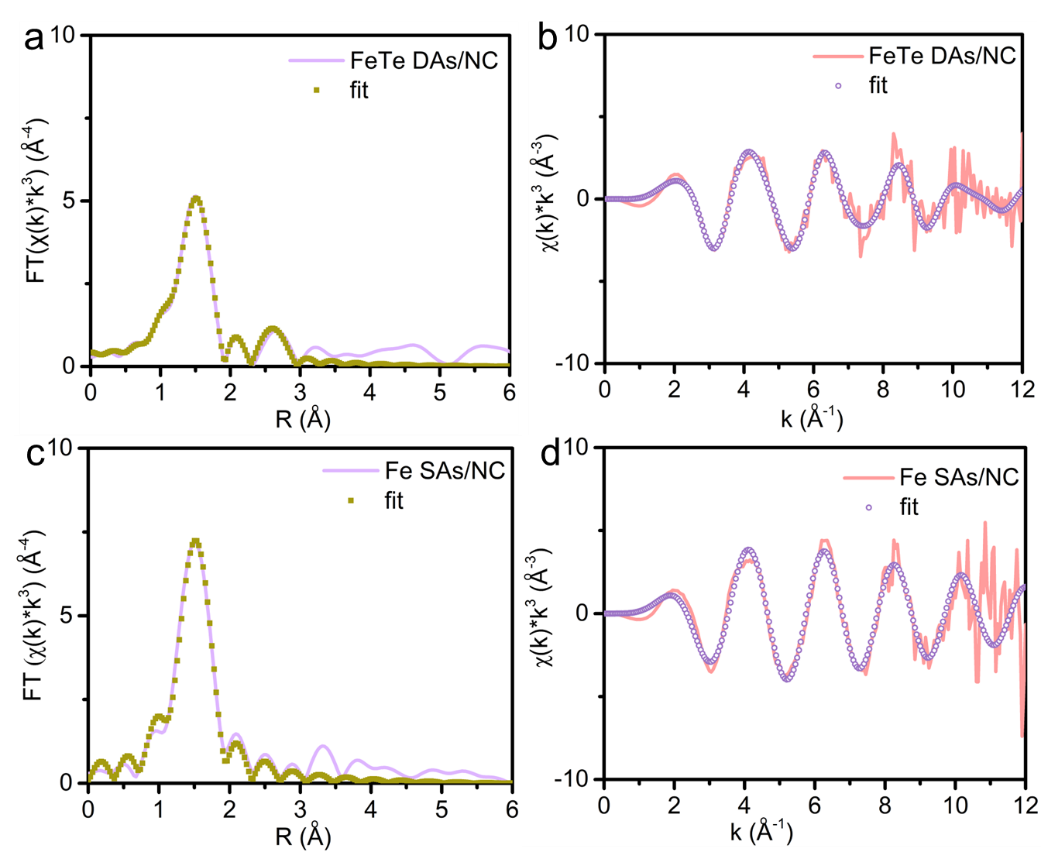


**Figure S19.** Experimental and fitting EXAFS curves of Fe k-edge in (a, c) R-space and (b, d) k-space for (a, b) FeTe DAs/NC and (c, d) Fe SAs/NC.


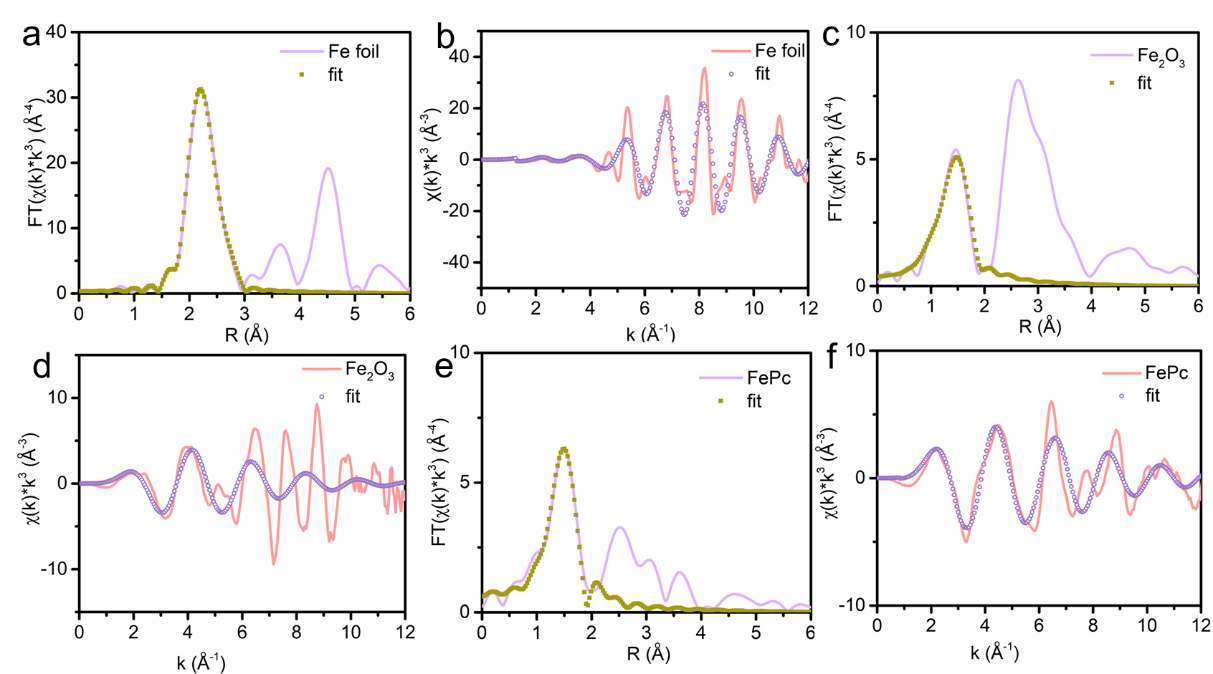


**Figure S20.** Experimental and fitting EXAFS curves of Fe k-edge in (a, c, and e) R-space and (b, d, and f) k-space for (a, b) Fe foil, (c, d) Fe_2_O_3_, and (e, f) FePc.


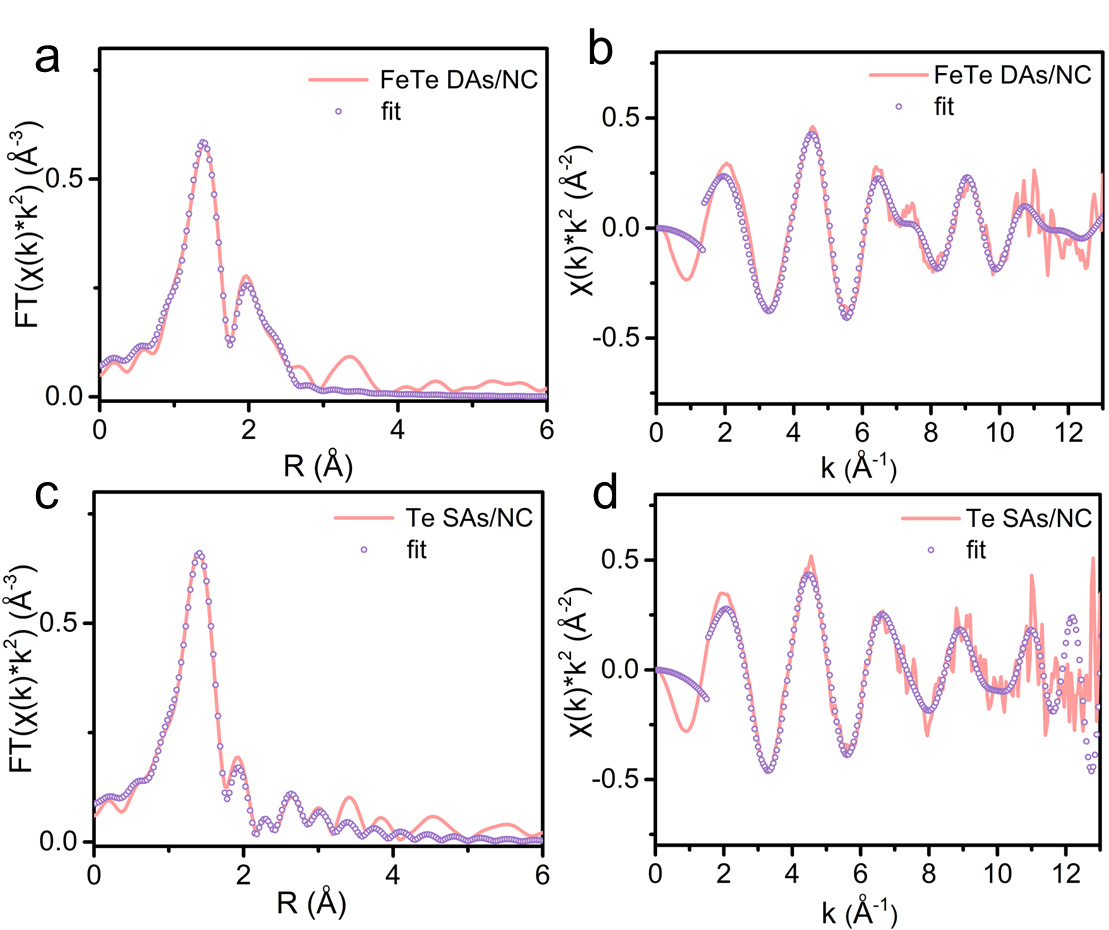


**Figuer S21.** Experimental and fitting EXAFS curves of Te k-edge in (a, c) R-space and (b, d) k-space for (a, b) FeTe DAs/NC and (c, d) Te SAs/NC.


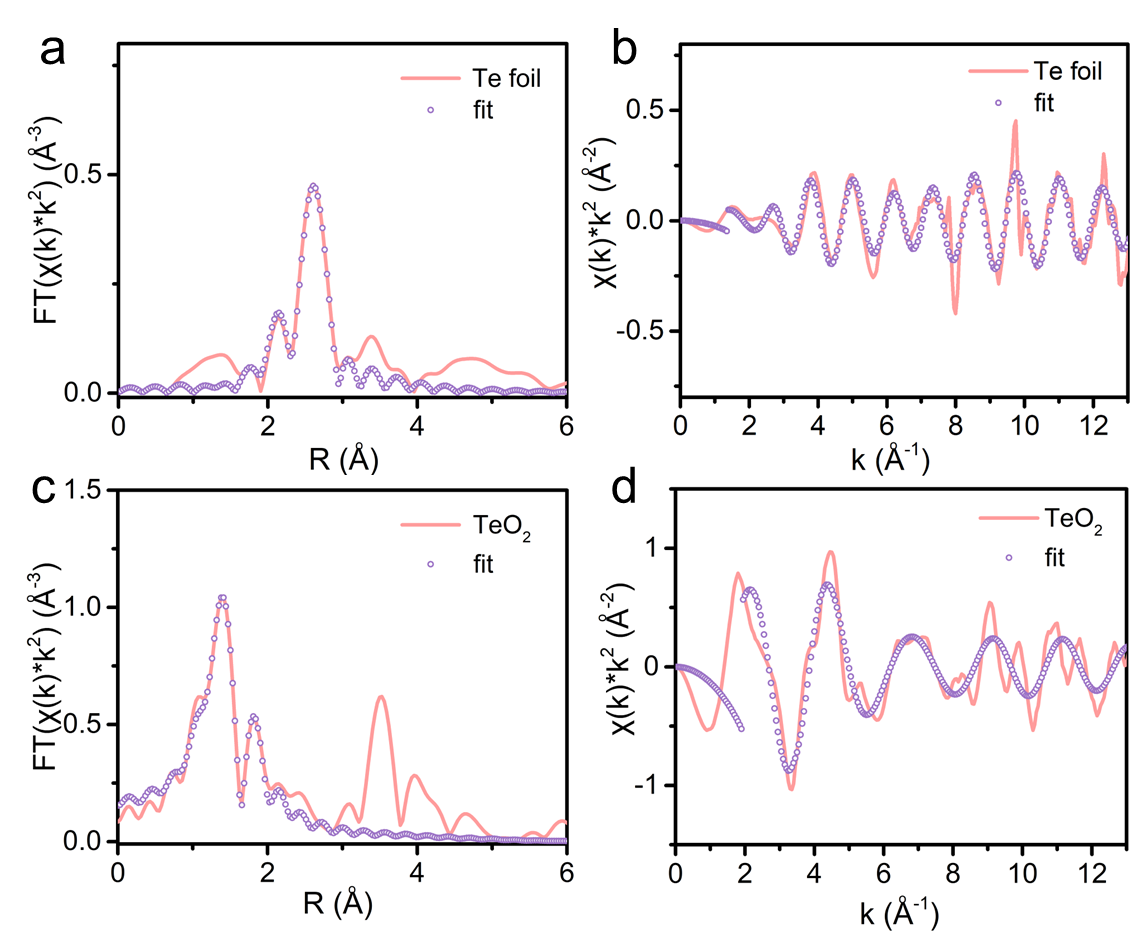


**Figure S22.** Experimental and fitting EXAFS curves of Te k-edge in (a, c) R-space and (b, d) k-space for (a, b) Te foil and (c, d) TeO_2_.


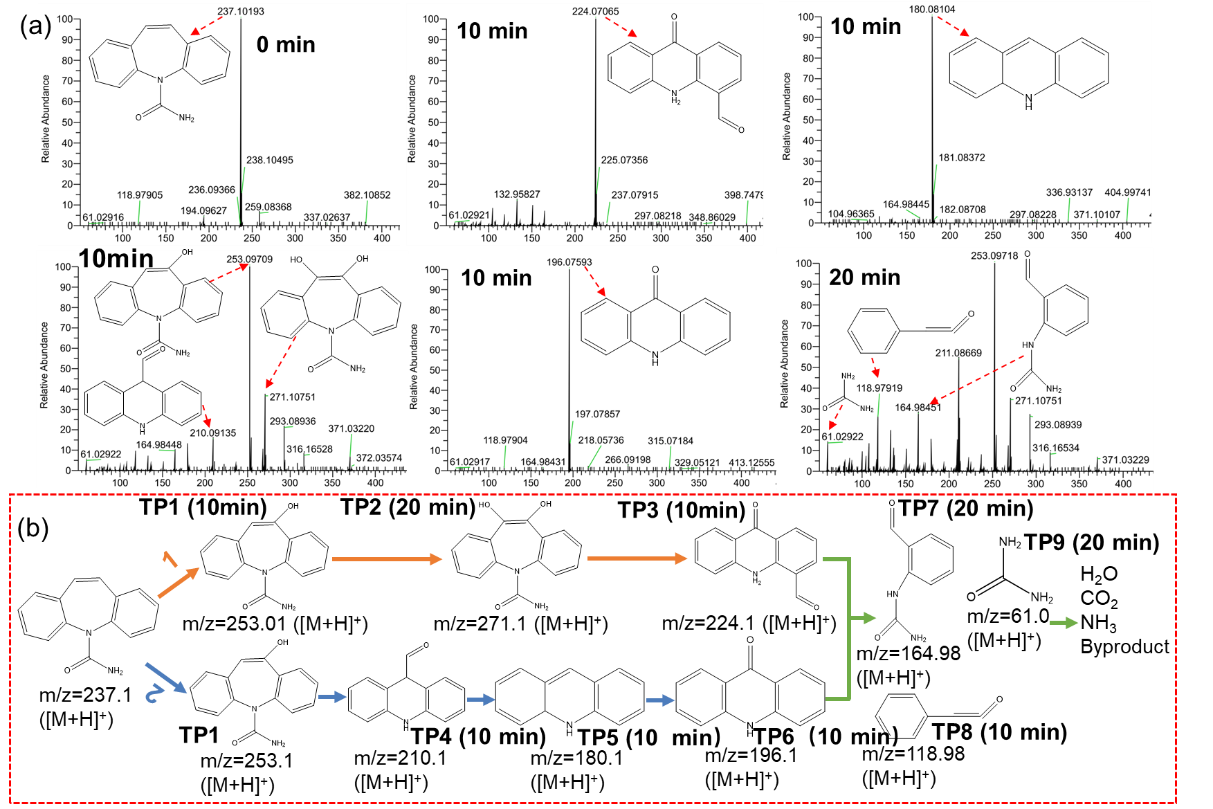


**Figure S23.** (a) LC-MS mass spectra of key intermediate products during CBZ degradation at different time intervals. (b) Proposed degradation pathway of CBZ based on the identified intermediates; 10 min and 20 min denote the first detection time of corresponding species.


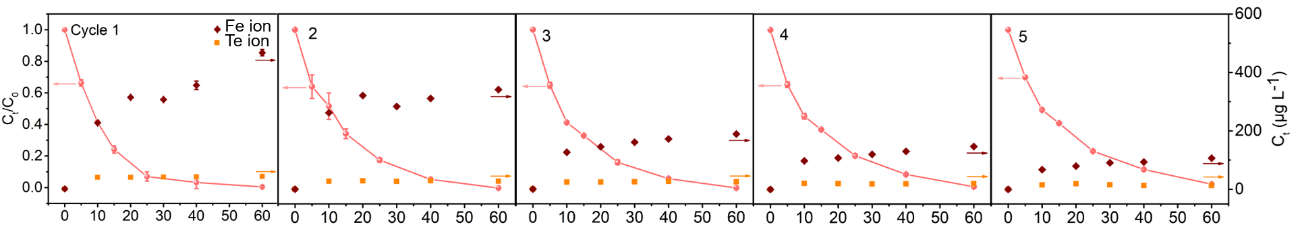


**Figure S24.** Cyclic CBZ degradation performance and the corresponding concentration of leaching Fe and Te ions. Reaction conditions: [catalyst] = 0.1 g L^−1^, [PMS] = 0.05 g L^−1^, [contaminant] = 10 mg L^−1^.





**Figure S25.** Cyclic CBZ degradation kinetic constants. Reaction conditions: [catalyst] = 0.1 g L^−1^, [PMS] = 0.05 g L^−1^, [contaminant] = 10 mg L^−1^.





**Figure S26.** Comparing the PXRD pattern of the used FeTe DAs/NC and FeTe DAs/NC.





**Figure S27.** Comparing the Raman spectra of the used FeTe DAs/NC and FeTe DAs/NC.





**Figure S28.** EPR spectra in the activation of PMS in the presence of Fe SAs/NC catalyst with DMPO in water.





**Figure S29.** EPR spectra in the activation of PMS in the presence of Te SAs/NC catalyst with DMPO in water.





**Figure S30.** EPR spectra in the activation of PMS in the presence of Fe SAs/NC catalyst with DMPO in methanol.





**Figure S31.** EPR spectra in the activation of PMS in the presence of FeTe DAs/NC catalyst with DMPO in methanol.





**Figure S32.** EPR spectra in the activation of PMS in the presence of Te SAs/NC catalyst with DMPO in methanol.





**Figure S33.** EPR spectra in the activation of PMS in the presence of Fe SAs/NC catalyst with TEMP in water.





**Figure S34.** EPR spectra in the activation of PMS in the presence of Te SAs/NC catalyst with TEMP in water.


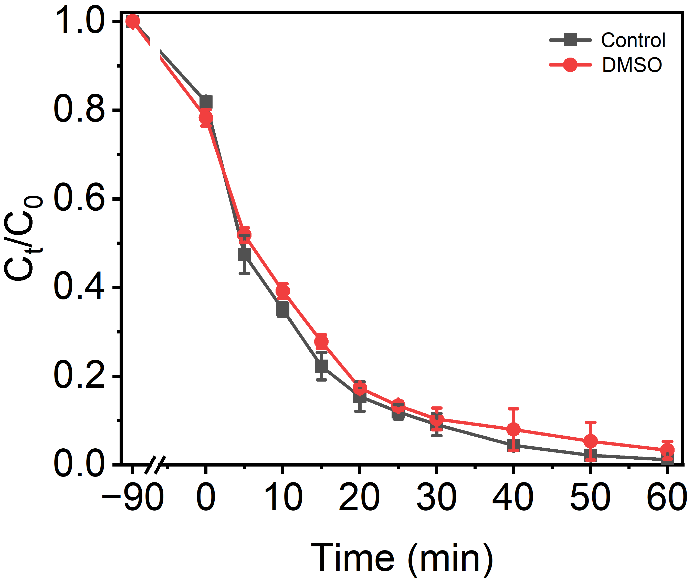


**Figure S35.** Quenching experiments (Dimethylsulfoxide, DMSO) for the degradation of CBZ in the FeTe DAs/NC-PMS system.





**Figure 36.** Quenching experiments for the degradation of CBZ in the Fe SAs/NC-PMS system. (Experimental conditions: 100 mL reaction solution, 10 mg L^-1^ pollutants, 2mM PMS, 100 mg L^-1^ catalyst, 6.0 pH).





**Figure 37.** Quenching experiments for the degradation of CBZ in the Te SAs/NC-PMS system. (Experimental conditions: 100 mL reaction solution, 10 mg L^-1^ pollutants, 2mM PMS, 100 mg L^-1^ catalyst, 6.0 pH).





**Figure S38.** **Chronoamperometry analysis of electron transfer behavior among different catalysts.** Chronoamperometry measurements of FeTe DAs/NC, Fe SAs/NC and Te SAs/NC catalysts upon sequential addition of PMS (peroxymonosulfate) and CBZ (carbamazepine).


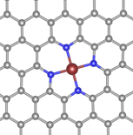




**Figure S39.** Partial density of states for (a) Fe of Fe SAs/NC (inset: atomic configuration of Fe-N_4_).


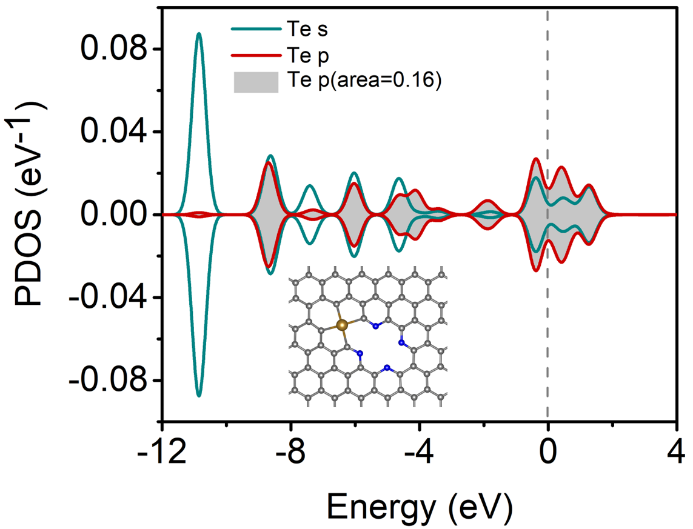


**Figure S40.** Partial density of states for Te of Te SAs/NC (inset: atomic configuration of Te-C_3_).

**Table S1.** ICP-MS results of the FeTe DAs/NC, Fe SAs/NC and Te SAs/NC catalysts.

| **Catalysts** | **Fe (wt. %)** | **Te (wt. %)** |
| --- | --- | --- |
| **FeTe DAs/NC** | 0.30 | 1.52 |
| **Fe SAs/NC** | 0.78 | _ |
| **Te SAs/NC** | _ | 4.09 |

**Table S2.** Fe K-edge EXAFS spectra fitting parameters.

| **Sample** | **Path** | **CN** | **R (Å)** | **σ^2^ (×10^-3^ Å^2^)** | **△E(eV** | **R (%)** |
| --- | --- | --- | --- | --- | --- | --- |
| **Fe foil^[a]^** | Fe-Fe_1_ | 8 | 2.47±0.01 | 4.8±1.0 | 6.25±1.67 | 0.5 |
|  | Fe-Fe_2_ | 6 | 2.85±0.01 | 5.9±1.8 |  |  |
| **Fe_2_O_3_^[b]^** | Fe-O | 6 | 1.97±0.03 | 12.9±4.6 | 3.95±3.13 | 0.9 |
| **FePc^[c]^** | Fe-N | 4 | 1.97±0.01 | 8.9±1.8 | 2.59±1.43 | 0.2 |
| **Fe SAs/NC^[d]^** | Fe-N | 4.3±0.5 | 1.99±0.01 | 3.9±1.8 | 3.17±1.48 | 0.3 |
| **FeTe DAs/NC^[e]^** | Fe-N | 3.3±0.3 | 1.97±0.01 | 7.0±1.4 | 1.26±0.92 | 0.1 |
|  | Fe-Te | 0.8±0.5 | 2.80±0.04 | 10.4±1.0 |  |  |

**Note:** N, coordination number; R, distance between absorber and backscatter atoms; σ^2^, Debye-Waller factor to account for both thermal and structural disorders; ΔE_0_, inner potential correction; R factor (%) indicates the goodness of the fit. S_0_^2^ was fixed to 0.72 as determined from Fe foil fitting.

[a] Fitting range: 3.0≤ k (/Å) ≤13.6 and 1.3≤ R (/Å) ≤2.9.

[b] Fitting range: 2.6≤ k (/Å) ≤11.3 and 1.0≤ R (/Å) ≤2.0.

[c] Fitting range: 2.7≤ k (/Å) ≤11.2 and 1.0≤ R (/Å) ≤1.9.

[d] Fitting range: 2.5≤ k (/Å) ≤10.6 and 1.0≤ R (/Å) ≤2.0.

[e] Fitting range: 2.5≤ k (/Å) ≤11.5 and 1.0≤ R (/Å) ≤2.3.

**Table S3.** Te K-edge EXAFS spectra fitting parameters.

| **Sample** | **Path** | **CN** | **R (Å)** | **σ^2^ (×10^-3^ Å^2^)** | **ΔE_0_ (eV)** | **R (%)** |
| --- | --- | --- | --- | --- | --- | --- |
| **Te foil^[a]^** | Te-Te | 2 | 2.83±0.01 | 5.5±0.7 | 6.98±0.65 | 0.8 |
| **TeO_2_^[b]^** | Te-O_1_ | 2 | 1.88±0.01 | 1.6±0.1 | 14.3±1.26 | 0.5 |
|  | Te-O_2_ | 2 | 2.10±0.03 | 6.6±2.2 |  |  |
| **Te SAs/NC^[c]^** | Te-C | 3.6±0.2 | 1.89±0.01 | 3.7±1.4 | 9.36±1.28 | 1.7 |
| **FeTe DAs/NC^[d]^** | Te-C | 3.1±0.1 | 1.88±0.01 | 3.6±1.0 | 7.15±1.12 | 0.4 |
|  | Te-Fe | 0.8±0.2 | 2.57±0.01 | 5.3±2.0 |  |  |

**Note:** N, coordination number; R, distance between absorber and backscatter atoms; σ^2^, Debye-Waller factor to account for both thermal and structural disorders; ΔE_0_, inner potential correction; R factor (%) indicates the goodness of the fit. S_0_^2^ was fixed to 0.77 as determined from Te foil fitting.

[a] Fitting range: 2.7≤ k (/Å) ≤12.6 and 1.9≤ R (/Å) ≤3.0.

[b] Fitting range: 2.7≤ k (/Å) ≤13.7 and 1.0≤ R (/Å) ≤2.0.

[c] Fitting range: 2.6≤ k (/Å) ≤11.6 and 1.0≤ R (/Å) ≤2.4.

[d] Fitting range: 2.6≤ k (/Å) ≤11.7 and 1.0≤ R (/Å) ≤2.6.

**Table S4.** Different Pollutants of degradation removal efficiency and rate constants in the FeTe DAs/NC system with PMS.

| **Pollutant** | **SMX** | **SSM** | **NFC** | **OFC** | **DF** | **IBU** |
| --- | --- | --- | --- | --- | --- | --- |
| **Removal efficiency (%)** | 96.2 | 97.1 | 93.6 | 91.6 | 99.3 | 52.1 |
| **Rate constant (min^-1^)** | 0.0411 | 0.0411 | 0.0291 | 0.0809 | 0.0859 | 0.0094 |

**Table S5.** The values of equivalent circuit elements fitting from EIS spectra.

| **Element** | **R_s_** | **CPE** | **R_1_** | **Q-Y_o_** | **Q-n** | **R_2_** |
| --- | --- | --- | --- | --- | --- | --- |
| **Fe SAs/NC** | 21.3 | 8.79×10^-6^ | 7.42×10^4^ | 6.47×10^-4^ | 0.75 | 12.4 |
| **FeTe DAs/NC** | 2.07 | 3.70×10^-4^ | 1.73×10^3^ | 6.47×10^-3^ | 0.81 | 3.41 |
| **Te SAs/NC** | 2.16 | 1.21×10^-4^ | 5.16×10^3^ | 1.55×10^-3^ | 0.75 | 4.16 |

**References:**

[1] J. Hutter, M. Iannuzzi, F. Schiffmann, J. VandeVondele, Wiley Interdiscip.Rev. Comput. Mol. Sci. 2014, 4, 15-25.

[2] J. VandeVondele, J. Hutter, J. Chem. Phys. 2007, 127, 114105.

[3] S. Goedecker, M. Teter, J. Hutter, J. Chem. Phys. 1996, 54, 1703.

[4] S. Grimme, J. Antony, S. Ehrlich, H. Krieg, J. Chem. Phys. 2010, 132, 154104.

[5] A.D. Becke, Phys. Rev. A 1988, 38, 3098.

[6] C. Lee, W. Yang, R.G. Parr, Phys. Rev. B 1988, 37, 785.

[7] M. Massucci, S.L. Clegg, P. Brimblecombe, J. Chem. Eng. Data 1996, 41, 765-778.

[8] J.J. Marti, A. Jefferson, X.P. Cai, C. Richert, P.H. McMurry, F. Eisele, J. Geophys. Res-Atmos. 1997, 102, 3725-3735.
